# Deepfakes and scientific knowledge dissemination

**DOI:** 10.1038/s41598-023-39944-3

**Published:** 2023-08-18

**Authors:** Christopher Doss, Jared Mondschein, Dule Shu, Tal Wolfson, Denise Kopecky, Valerie A. Fitton-Kane, Lance Bush, Conrad Tucker

**Affiliations:** 1 RAND Corporation, Santa Monica, USA; 2 Carnegie Mellon University, Pittsburgh, USA; 3 Pardee RAND Graduate School, Santa Monica, USA; 4 Challenger Center, Washington, D.C. USA

**Keywords:** Governance, Computer science

## Abstract

Science misinformation on topics ranging from climate change to vaccines have significant public policy repercussions. Artificial intelligence-based methods of altering videos and photos (deepfakes) lower the barriers to the mass creation and dissemination of realistic, manipulated digital content. The risk of exposure to deepfakes among education stakeholders has increased as learners and educators rely on videos to obtain and share information. We field the first study to understand the vulnerabilities of education stakeholders to science deepfakes and the characteristics that moderate vulnerability. We ground our study in climate change and survey individuals from five populations spanning students, educators, and the adult public. Our sample is nationally representative of three populations. We found that 27–50% of individuals cannot distinguish authentic videos from deepfakes. All populations exhibit vulnerability to deepfakes which increases with age and trust in information sources but has a mixed relationship with political orientation. Adults and educators exhibit greater vulnerability compared to students, indicating that those providing education are especially susceptible. Vulnerability increases with exposure to potential deepfakes, suggesting that deepfakes become more pernicious without interventions. Our results suggest that focusing on the social context in which deepfakes reside is one promising strategy for combatting deepfakes.

## Introduction

Widespread use of the internet by learners of all ages has democratized the development and accessibility of educational materials^1^. The COVID-19 pandemic further solidified digital communication as a primary medium for information exchange among educators and learners^2^. Indeed, K-12 students are digital natives who use various online platforms such as YouTube to complete academic assignments^3^, with varying degrees of judgement for the reliability of the sources^4^. The amount of time children eight years old or less spent on YouTube has doubled between 2017 and 2020, portending an increased reliance on online media for even the youngest learners^5^. Social media and other online information sources have also facilitated lifelong education for continued professional and personal development^6^. However, the proliferation of misinformation on social media and other platforms raises the risk of exposure to deliberately misleading educational content^7,8^.

The consequences of exposure to online misinformation range in severity and scale, often depending on context^9^. While many types of misinformation exist outside of the mainstream^10^, misinformation on science, technology, engineering, and math (STEM) related topics such as climate change and vaccines have had major public policy repercussions^11–14^. Social media and modern communication methods facilitate the rapid dissemination of misinformation, amplifying these impacts^15,16^. Misinformation campaigns tend to rely on undermining the consensus, highlighting uncertainty, undermining the credibility of leading figures and institutions, and disseminating pseudoscientific alternatives^17^.

The availability of open-source artificial intelligence (AI) algorithms has significantly lowered the barriers to altering videos and images in order to produce highly realistic, manipulated digital content (e.g., deepfakes)^18–20^. Generative neural networks (GNNs) are a class of deep neural network models that represent the state-of-the-art technique that can be leveraged to democratize the mass synthesis of manipulated digital content^21,22^. They have been used to fabricate images by training them to encode human features^23^, to manipulate images via replacing specific components of a digital image or video^24^, and to create videos via animation of a still image with the characteristics of a source video^25^.

In this study, we investigate the vulnerabilities of K-12 students, higher education students, teachers, principals, and general adult learners to deepfakes related to climate change and investigate potential population and video characteristics that can be leveraged in mitigation approaches. To date, the anticipated prevalence of deepfakes across societal contexts has motivated a large body of work seeking to develop algorithmic techniques to detect deepfakes^26–37^. However, these algorithms exhibit low rates of successful detection and are not robust across deepfake types, content format, content characteristics, and datasets^20,38^. Parallel efforts to advance user-focused solutions are nascent and characterized by high failure rates^39^. Recent work indicates that human–machine teams show promise for overcoming these challenges to identifying deepfakes^40–42^; these studies, however, don’t account for the social and individual characteristics that modulate individuals’ vulnerabilities to deepfakes. The successful design, development, and deployment of human–machine teams for deepfake detection and other mitigation strategies requires a comprehensive understanding of individuals’ abilities to successfully detect deepfakes, personal characteristics that moderate individuals’ vulnerability to deepfakes, and digital content characteristics that influence successful detection^43,44^. The enabling data, however, have not yet been made available.

The detection and mitigation against deepfakes are particularly needed within STEM education given increasing access to and reliance on readily available digital educational content by both youth and adult learners^45^. To date, work investigating the vulnerabilities of K-12 students to STEM misinformation has tended to focus on deliberately falsified text-based content and media literacy^46–50^. A limited number of studies have investigated adults’ vulnerability to deepfakes, but these have been limited to deepfakes depicting politicians, how it impacts voters’ attitudes toward politicians depicted in the videos, and how vulnerability can be moderated by personal characteristics (e.g., religious convictions, political orientation), as well as attempted inoculations within these contexts^41,51,52^ (Table A1 in Appendix A). Indeed, this research on politically-based deepfakes follows a robust line of research on the spread of political misinformation on social media during the 2016 U.S. presidential election^53^.

Climate change is a particularly compelling aspect of STEM to explore because the polarized nature of climate change has left this domain vulnerable to digital misinformation. Climate change misinformation outside of deepfakes is pervasive and typically relies on recipients’ motivated cognition to protect against ideologically or economically threatening scientific evidence^54,55^ to gain traction. Weak media literacy skills, particularly among K-12 students, has also been shown to moderate susceptibility^56^. Historically, producing convincing fabricated or manipulated digital content (data, videos, audio, etc.) related to climate change has been much more challenging^55^. However, the emergence of AI algorithms—particularly to create deepfakes—increases the risk of exposure to convincing climate change misinformation^57^. Additionally, it’s currently unknown if deepfakes present novel threat vectors that can take advantage of similar vulnerabilities as mainstream climate change misinformation or if deepfakes expand the misinformation attack surface.

To investigate the vulnerabilities of the education system climate change deepfakes, we fielded a survey that embedded a series of randomly assigned authentic or deepfake videos on climate change. We then asked respondents to identify the video as authentic or manipulated and gathered information regarding respondents’ demographics, background knowledge of climate change, learning habits, and perspectives on deepfakes. We found that between 27 percent to over half of survey responses were unable to correctly identify the authenticity of videos *regardless* of whether the video was authentic or a deepfake. In aggregate, U.S. adults and educators were less likely to correctly identify deepfake videos than authentic videos, while middle school and higher education students were more likely to identify deepfake videos than authentic videos. However, vulnerability fluctuates across individual deepfake videos and can be quite severe. Heterogeneity analyses indicated that an individual’s susceptibility varies as a function of age, political orientation, and trust in information sources. Further, vulnerabilities increased dramatically as individuals were exposed to more potential deepfakes, suggesting that deepfakes can become more pernicious without educational interventions. An analysis of video characteristics that respondents reported drove their decisions indicated that the social context in which deepfakes are embedded could provide a promising approach for educational mitigation strategies. We conclude by discussing the implications of these results on the development of technical and social mitigation strategies for combatting STEM-focused deepfakes.

## Survey instrument and sample

We fielded our survey to five key populations: nationally representative samples of (1) adults in the U.S. 18 years of age and older, (2) U.S. K-12 teachers, and (3) U.S. K-12 principals, (4) a large sample of middle school students from three states across the U.S., and (5) a sample of undergraduate and graduate students at Carnegie Mellon University (CMU). See methods section for more detail on these samples.

Our main research question, which sought to understand the vulnerability of each population to the deepfake videos, is:

1. What is the effect of receiving a deepfake video on an individual’s ability to correctly identify a video’s authenticity and how does that effect vary by population?
  Our secondary research questions, which leverage the contextual questions in the surveys to uncover drivers of vulnerabilities and potential avenues for deepfake mitigation, were as follows:
2. What characteristics of the video do respondents analyze to determine the authenticity of the video and how are they related to the probability of correctly identifying the video’s authenticity?
3. How does the effect of a deepfake video on an individual’s ability to correctly identify a video’s authenticity vary by respondent background characteristics and beliefs?

The survey instrument took about ten minutes to complete and was divided into two portions. One portion presented the respondent with four videos, each about 10–15 s in length. Each video featured one of four speakers: (1) Timothy Gallaudet, an oceanographer and former Acting Administrator of the National Oceanic and Atmospheric Administration; (2) Richard Lindzen, Professor Emeritus of Meteorology at the Massachusetts Institute of Technology; (3) Greta Thunberg, noted climate change activist; and (4) Naomi Seibt, anti-climate change activist. These speakers were chosen to expose respondents to a variety of views and types of speakers on climate change that youth and adult learners are likely to encounter. Gallaudet and Lindzen are credentialed speakers with positions of scientific authority with opposing views on climate change. While Gallaudet is a climate change believer, Lindzen is a climate change skeptic. Greta Thunberg and Naomi Seibt are both younger activists and represent the climate change and anti-climate change perspective, respectively.

The four videos were drawn from a bank of eight possible videos. For each speaker, the bank contained an authentic video in which the speaker espoused their view of climate change and an AI generated deepfake video where the speaker is made to espouse their opposite view of climate change. Thus, climate change believers are made to be climate change skeptics and vice versa. Each respondent received one video from each speaker and was randomly assigned to receive an authentic or deepfake video of each speaker. A participant could view an authentic video of one speaker, but a deepfake of another speaker. In contrast to prior studies^41^, we did not tell participants the probability of being exposed to a deepfake because stakeholders would not be privy to such information when encountering deepfakes in real-life. We randomized the order of the speakers for each respondent to avoid any effects of being exposed to prior authentic or deepfake videos.

The second portion of the survey elicited the views of the respondents on a variety of dimensions including: personal climate change beliefs, perceptions of the scientific consensus on the causes of climate change, knowledge of climate change, political affiliation (asked only of adults), information sources respondents use to learn about climate change, trust in those information sources, internet use habits, use of social media platforms, perceptions of the risks associated with and the prevalence of deepfakes, and demographic questions.

## Results

### The ability of respondents to correctly detect deepfake and authentic videos

**Figure 1:**
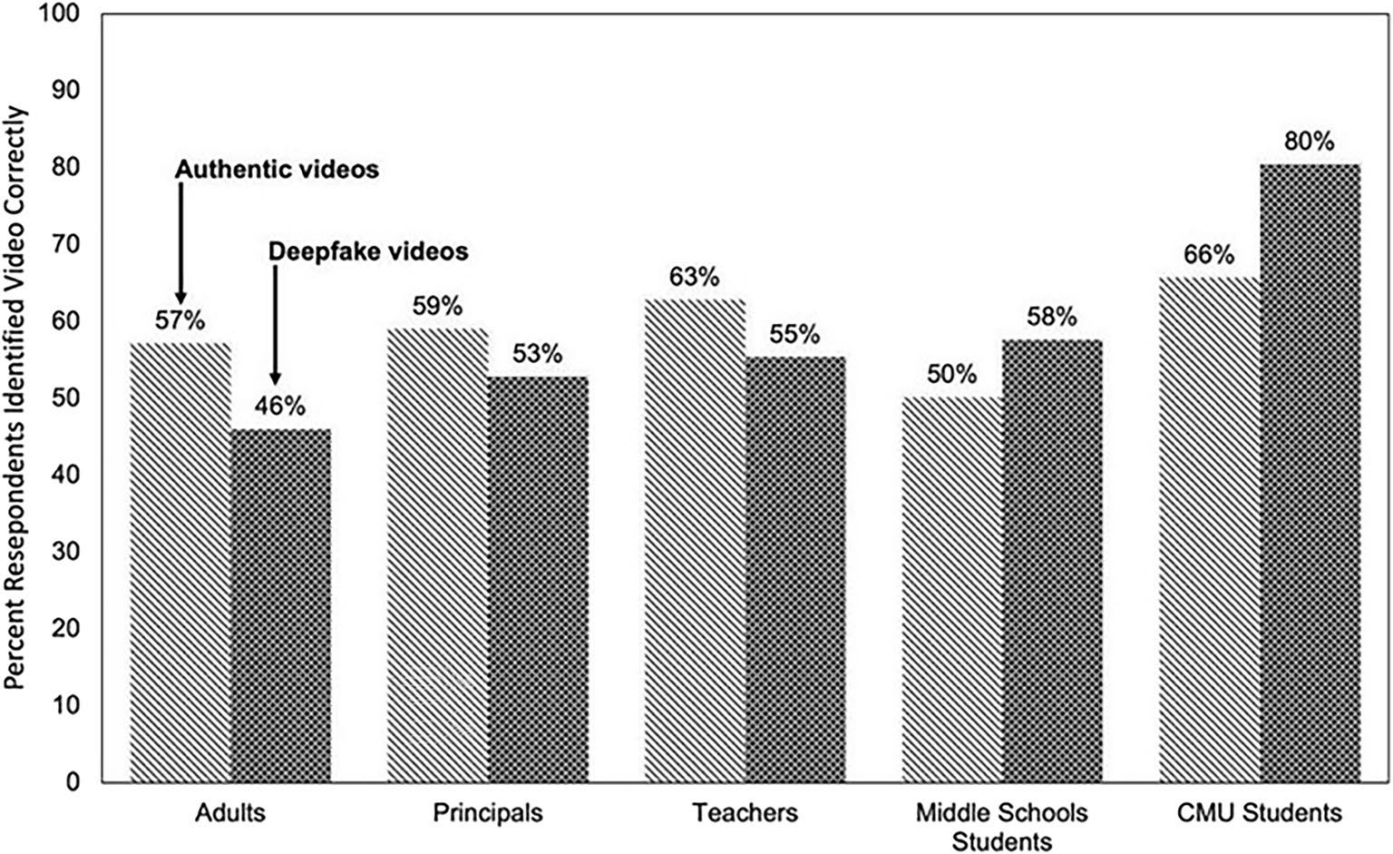
Percent of Responses Correctly Identifying the Authenticity of Videos, by Video Authenticity and Population. *Notes*: Each bar represents the percentage of responses that correctly identified the authenticity of videos by population and deepfake video status. Tabulations in the adult, principal, and teacher populations are weighted to be nationally representative.

Across all videos, substantial percentages of respondents did not correctly identify a video’s authenticity when receiving deepfake *or* authentic videos (Fig. 1). When receiving authentic videos, the percentage of correct responses ranged from 50 percent (middle school students) to 66 percent (CMU students). When receiving deepfake videos, the percentage of correct responses ranged from 46 percent (adults) to 80 percent (CMU students). Figure A1 in Appendix A shows that among the responses that were not correct, a substantial number of responses were either “Cannot Tell” (between 10 and 20 percent of responses) or incorrect (between 11 and 33 percent of responses). Averaging correct responses across all videos within a population reveals that about 27–50% of education stakeholders were unable to correctly identify the authenticity of a video. These vulnerabilities to climate change-related deepfakes are similar to those seen in politically oriented deepfakes focused on elections and context-agnostic deepfakes^41,51,52^, indicating that vulnerabilities to deepfakes may be consistent across contexts. These rates also closely mirror the susceptibility rates of individuals to digital images altered manually without artificial intelligence^41,58^.

Our results further indicate that across all videos, receiving a deepfake lowered the probability that adults and educators correctly identified the authenticity of the video by 6 percentage points (principals) to 11 percentage points (adults) compared to when they received an authentic video. Based on the percentage of respondents that correctly identified authentic videos, these estimates represent a 10 percent (principals) to 19 percent (adults) decrease in correct responses. In contrast, student populations were better able to detect deepfake videos (compared to authentic videos) with an 8 percentage point increase in correct responses among middle school students and a 14 percentage point increase in correct responses from CMU students. This translated to a 16 and 21 percent increase in correct responses, respectively. Regression models presented in Table A2 in Appendix A show that these differences were statistically significant to the one percent level. Further, the effects of receiving a deepfake on the probability of responding “Cannot Tell” were small and statistically insignificant, implying that the deepfake videos induced participants to respond incorrectly in the adult and educator populations or correctly in the student populations.

These overall results, however, hide important heterogeneity as respondents’ accuracy in correctly identifying deepfake and authentic videos varied across individual videos (Figs. A2–A4 in Appendix A). CMU students were the only population more likely to correctly identify deepfake videos than the authentic videos across all speakers, likely because of their experience and expertise in artificial intelligence and machine learning. All other populations were less able to correctly identify at least two deepfake videos. Adults, teachers, and principals, and middle school students were less able to detect deepfake videos of Timothy Gallaudet and Richard Lindzen. The largest gap was observed for the U.S. adults that viewed the Richard Lindzen video. Adults were 39 percentage points less likely to have correctly identified the deepfake video compared to the authentic video. This gap is a nearly a 50 percent decline in the proportion of correct responses compared to the authentic video. Results for the Greta Thunberg and Naomi Seibt videos were mixed, with middle school students more likely to detect deepfakes of both speakers while adults, teachers, and principals were equally or less able to detect the deepfakes of these speakers. Table A3 in Appendix A shows that these effects were highly significant in most cases. More research needs to be done to understand the drivers of this heterogeneity in effects, though overall our results show that no population in our study was immune to deepfake video deception.

### Video characteristics moderating respondents’ vulnerability to deepfakes

Identifying the characteristics of deepfake videos that moderate an individuals’ vulnerability is an important step towards developing countermeasures. Understanding which aspects of a video respondents used in their decision-making process and how that differed by video authenticity provides an understanding of the respondents’ cognitive processes. For example, if receiving a fake video resulted in greater (or less) use of technical aspects of the video such as facial features or overall quality, then respondents are making decisions in part based on overall or specific flaws in the video. Similarly, if receiving a fake video resulted in a greater (or less) use of social aspects of the video such a familiarity with the speaker’s views or overall credibility of the content, then respondents are using contextual knowledge in their decision-making process. We then explore whether leveraging these aspects of videos are associated with the correct identification of authentic and manipulated videos.

The results in Table 1 indicate that respondents tended to use visual aspects of deepfake videos (e.g., facial features, video background, overall video quality) more often and social aspects of the video (e.g., credibility of the information being shared) or the audio quality less often when presented a deepfake. Principals, teachers, and middle school students were statistically significantly more likely to assess the overall quality of deepfake videos than authentic videos, while both teachers and CMU students were more likely to examine the facial features presented in deepfakes.

**Table 1:** Relationship between reported aspect of video analyzed and receiving deepfake video.

	Adults	Principals	Teachers	Middle school students	CMU students
Familiar with person's views					
	− 0.036	− 0.027 +	− 0.027 +	− 0.017	0.036
	(0.024)	(0.015)	(0.015)	(0.013)	(0.039)
Video quality					
	0.051	0.086**	0.074**	0.047*	0.034
	(0.034)	(0.023)	(0.023)	(0.020)	(0.062)
Background					
	0.008	0.002	− 0.011	− 0.017	0.048
	(0.024)	(0.020)	(0.020)	(0.019)	(0.055)
Facial features					
	0.011	0.009	0.048*	0.035 +	0.115*
	(0.035)	(0.021)	(0.022)	(0.019)	(0.047)
Audio					
	− 0.047	− 0.039*	− 0.087**	− 0.045*	− 0.185*
	(0.033)	(0.020)	(0.020)	(0.020)	(0.072)
Credibility of content					
	− 0.059*	− 0.101**	− 0.065**	− 0.048**	− 0.017
	(0.026)	(0.022)	(0.022)	(0.017)	(0.058)
N (Respondent-by-video)	3044	2,960	2,568	3,220	348
N (Respondent)	761	740	642	805	87

Each cell presents the results of a separate regression of whether a respondent reported analyzing an aspect of the video and receiving a fake video. All models include speaker, video order, and respondent fixed effects. Regressions models used to analyze adult, principal, and teacher panels are weighted to retain national representation. Standard errors are clustered by respondent.

+ indicates *p* < 0.10; *p < 0.05; **p < 0.01.

The results in Table 2 indicate the relationship between using these video aspects in the decision-making process on the likelihood of correctly identifying a video’s authenticity. These results indicate using a videos’ overall quality and the speaker’s facial features in the decision-making process is associated with a significantly increased probability of correctly identifying deepfake videos, but not authentic videos. Adults, principals, and teachers were 20–25% points more likely to accurately identify deepfakes when assessing the video quality while principals, teachers, and middle school students were 13–20% points more likely to correctly identify deepfakes when analyzing facial features. Analyzing the audio quality of the videos was not associated with higher rates of correctly identifying deepfakes videos but did lead to 13–22% point improvements in correctly identifying authentic videos by adults, principals, and middle school students. Thus, the usage of visual aspects of a video in the decision-making process when receiving a deepfake reduced viewers’ vulnerability. This finding is aligned with previous work that similarly found that focusing on the visual aspects of videos can reduce the susceptibility of adults to deepfakes^59^. Indeed, recent work has indicated that cognitive processing of faces is a helpful technique towards assessing the authenticity of visual media^41^.

**Table 2:** Relationship between reported aspect of video analyzed and correctly identifying video authenticity, by video deepfake status.

	Adults		Principals		Teachers		Middle school students		CMU students	
	Real	Deep fake	Real	Deep fake	Real	Deep fake	Real	Deep fake	Real	Deep fake
Familiar with person's views	0.381**	0.217 +	0.479**	0.223**	0.389**	0.101	0.409**	− 0.136	0.455*	0.044
	(0.089)	(0.118)	(0.069)	(0.082)	(0.071)	(0.085)	(0.067)	(0.084)	(0.199)	(0.192)
Video quality	− 0.128	0.251*	− 0.062	0.261**	− 0.134*	0.210**	0.020	0.085 +	− 0.059	− 0.124
	(0.081)	(0.097)	(0.049)	(0.058)	(0.058)	(0.059)	(0.049)	(0.052)	(0.147)	(0.139)
Background	− 0.135	0.088	− 0.075	0.083	0.010	− 0.023	0.003	0.126*	− 0.135	− 0.075
	(0.125)	(0.102)	(0.053)	(0.070)	(0.062)	(0.076)	(0.046)	(0.055)	(0.152)	(0.156)
Facial features	− 0.033	0.152 +	0.063	0.154**	0.011	0.133*	0.090	0.197**	− 0.011	− 0.154
	(0.069)	(0.082)	(0.047)	(0.057)	(0.058)	(0.061)	(0.056)	(0.055)	(0.163)	(0.134)
Audio	0.222**	− 0.001	0.222**	− 0.085	0.240**	− 0.102	0.133*	− 0.110*	0.152	− 0.074
	(0.076)	(0.084)	(0.052)	(0.058)	(0.060)	(0.065)	(0.054)	(0.051)	(0.163)	(0.123)
Credibility of content	0.157*	− 0.043	0.179**	− 0.073	0.064	− 0.091	0.093 +	− 0.084	0.214	− 0.065
	(0.063)	(0.074)	(0.050)	(0.057)	(0.057)	(0.056)	(0.056)	(0.067)	(0.131)	(0.153)
N (Respondent-by-video)	1600	1444	1514	1446	1283	1285	1600	1620	169	179
N (Respondent)	727	693	692	685	604	596	757	755	82	84

Each column presents the results of a separate regression of video authenticity on aspects respondents reported analyzing when making their decisions. All models include speaker, video order, and respondent fixed effects. Regressions models used to analyze adult, principal, and teacher panels are weighted to retain national representation. Standard errors are clustered by respondent.

+ indicates *p* < 0.10; *p < 0.05; **p < 0.01.

However, Table 2 also shows that propensity to use social aspects of the video less when receiving a deepfake video may not always be optimal, particularly for adults. Both adults and teachers that reported using their familiarity with the speaker’s views in their decision-making process were more likely to correctly identify deepfakes by about 22 percentage points (*p* < 0.10 for adults and *p* < 0.05 for teachers). Using the credibility of the content also helped adults and teachers accurately identify authentic videos. Respondents who reported that they were familiar with the speaker’s views were also statistically significantly more likely to correctly identify authentic videos in every population and with estimates that range from 38 to 48 percentage points. Educating information consumers to assess the social context that surround deepfakes such as content credibility and the speaker identity therefore present new focal points for strategies that may be more robust than a focus on technical aspects. This finding aligns with recent developments in the science of communication/misinformation fields, as higher levels of a priori knowledge has been found to increase cognitive reflection and decrease susceptibility to misinformation^17,60^. Indeed, over the long term the value of assessing the technical aspects of deepfake videos is likely to decline as deepfake generation technologies are expected to continue their rapid advancement towards producing content that is indistinguishable from authentic videos^61^.

### Respondent characteristics moderating vulnerability to deepfakes

The design of the survey instruments fielded in this work enabled an understanding of how respondent characteristics and beliefs moderated their vulnerabilities to deepfakes. Table 3 shows those characteristics that most consistently moderated affects across populations, while Table A6 in Appendix A shows that factors such as respondents’ race, beliefs in climate change, perceived ability to detect deepfakes, perceived risk of deepfakes, climate change knowledge, frequency of consumption of information, frequency of social media use, urbanicity, working in or teaching a science or math related field, income, and education did not consistently moderate ability to detect video authenticity across populations.

**Table 3:** Moderation of participant background characteristics on probability of correctly identifying deepfake video.

	Adults		Principals		Teachers		Middle school student		CMU students	
Panel A: Age in years										
Age	− 0.006*	− 0.004*	− 0.003	− 0.003	− 0.005*	− 0.006**	− 0.019	− 0.035*	− 0.029	− 0.032
	(0.003)	(0.002)	(0.003)	(0.003)	(0.002)	(0.002)	(0.020)	(0.015)	(0.044)	(0.037)
Panel B: Political orientation (Reference category: liberal)										
Conservative	− 0.151 +	− 0.150*	− 0.096	− 0.097*	0.120 +	0.132**	–	–	0.076	0.010
	(0.087)	(0.065)	(0.062)	(0.047)	(0.062)	(0.045)	–	–	(0.193)	(0.187)
Moderate	− 0.077	− 0.062	− 0.126*	− 0.116*	0.145*	0.166**	–	–	0.033	0.008
	(0.106)	(0.073)	(0.058)	(0.046)	(0.066)	(0.051)	–	–	(0.184)	(0.155)
Prefer not To say	− 0.095	− 0.080	0.125	0.081	0.021	0.077	–	–	− 0.158	− 0.220 +
	(0.128)	(0.101)	(0.091)	(0.068)	(0.080)	(0.063)	–	–	(0.147)	(0.131)
Panel C: Trust in information sources										
Trust	− 0.043	− 0.022	− 0.034	− 0.046*	− 0.043	− 0.048*	− 0.071**	− 0.049**	− 0.094 +	− 0.119*
	(0.029)	(0.024)	(0.027)	(0.021)	(0.026)	(0.020)	(0.024)	(0.019)	(0.055)	(0.054)
Panel D: Order of videos seen (Reference category: first video)										
Second video	0.066	0.026	− 0.121 +	− 0.112*	− 0.096	− 0.098 +	− 0.110 +	− 0.084	− 0.265 +	− 0.315*
	(0.104)	(0.079)	(0.064)	(0.050)	(0.075)	(0.056)	(0.067)	(0.051)	(0.150)	(0.121)
Third video	0.070	0.031	− 0.170**	− 0.143**	− 0.192*	− 0.215**	− 0.129*	− 0.179**	− 0.357*	− 0.472**
	(0.110)	(0.082)	(0.064)	(0.051)	(0.075)	(0.056)	(0.063)	(0.048)	(0.170)	(0.133)
Fourth video	0.026	0.019	− 0.149*	− 0.138**	− 0.126 +	− 0.175**	− 0.222**	− 0.223**	− 0.257	− 0.352**
	(0.110)	(0.084)	(0.064)	(0.051)	(0.070)	(0.053)	(0.063)	(0.048)	(0.155)	(0.114)
Respondent fixed effects	**✓**		**✓**		**✓**		**✓**		**✓**	
Respondent controls		**✓**		**✓**		**✓**		**✓**		**✓**
N (Respondent-by-video)	3044	2960	2568	3220	348					
N (Respondent)	761	740	642	805	87					

Each cell presents the interaction term of a separate regression of whether a respondent correctly identified the authenticity of a video on the main effect for seeing a deepfake video and an interaction with the characteristic indicated by the row headers. All models include speaker and video order fixed effects. Models include respondent fixed effects or respondent controls as indicated in the table. In all models, respondent controls are panel specific variables listed in Table 4. The middle school and CMU student samples also include state fixed effects. Regressions models used to analyze adult, principal, and teacher panels are weighted to retain national representation. Standard errors are clustered by respondent.

+ indicates *p* < 0.10; *p < 0.05; **p < 0.01.

In both the general adult population and the teacher population, the ability to detect deepfake videos declined with age. We found that each year of age was associated with a 0.6 percentage point reduction in likelihood of correctly identifying deepfakes (Panel A of Table 3). Similar trends were observed for the principal, middle school, and CMU panels but these relationships were not as robustly statistically significant across specifications. This finding contrasts with earlier work that found vulnerability to digital misinformation decreased as a function of age^62–64^. The digital misinformation in these studies did not include deepfakes, which indicates that deepfakes present a distinct modality of misinformation that affects information consumers differently than other misinformation pathways. While further work is required to better understand the mechanisms driving the higher susceptibility of older individuals, related work indicates that this increased vulnerability may be due to higher degrees of trust and greater difficulty detecting lies, in addition to a lower familiarity with social media^65^. These results suggest that older populations should be prioritized as target audiences for deepfake mitigation strategies. This is the first study identifying higher levels of vulnerability to deepfakes among older information consumers.

The relationship between vulnerability to deepfakes and respondents’ political orientation also indicates that deepfakes may be considered a distinct type of misinformation. Similar to the extant literature that indicates individuals who identify as politically conservative tend to be more vulnerable to climate change misinformation^64,66^, we found that self-identified conservatives were less likely than self-identified liberals to correctly identify deepfakes by 10–15 percentage points in the adult and principal populations (Panel B of Table 3). However, among teachers, self-identified conservatives were better than liberals at identifying deepfakes by about 13 percentage points. The cause for this reduced vulnerability among self-identified politically conservative teachers is unknown but this finding indicates that exposure to deepfakes may not trigger identity protective cognition, a factor commonly associated with greater acceptance of climate change misinformation among politically conservative audiences^60^. Future work is needed to further understand how participant beliefs and perspectives across populations intersect with the content and social context of deepfakes to produce these more nuanced results.

However, similar to other modalities of STEM misinformation^67,68^, we found that an individual’s trust in information sources (e.g., social media, newspapers, etc.) influenced their vulnerability to deepfakes. Panel C of Table 3 shows that for principals, teachers, middle school students, and CMU students, a one standard deviation increase in our factor of information trust (see Appendix C for more information on the factor) reduced the probability of correctly identifying a deepfake by about 5–12 percentage points. A similar relationship was observed among U.S. adults, although this was not statistically significant in any specification. This finding suggests that media literacy approaches, which aim to combat misinformation by teaching information verification techniques^49,56^, may be a promising mitigation strategy to reduce vulnerability to deepfakes.

Our results also indicate that other mitigation strategies, such as training information consumers to identify deepfakes via repeated exposure, are unlikely to succeed and may worsen vulnerabilities (Panel D of Table 3). Among teachers, principals, middle school students, and CMU students, respondents were 13–47 percentage points less likely to correctly identify video authenticity for the third and fourth video consumed (no relationship between exposure and vulnerability was detected for U.S. adults). These results indicate that without education on deepfakes, consistent exposure to potential deepfakes may cause more confusion that leads to a worsening effect of deepfake misinformation.

## Conclusions

Society is at an inflection point regarding misinformation as social media platforms and the ubiquity of technology have allowed misinformation to proliferate and emerging technologies have reduced the barriers to production. STEM misinformation poses a substantial threat to society because it can impede policy reforms needed to combat global challenges such as climate change. To date, technological limitations meant that misinformation was usually disseminated in print, through the manipulation of still photos, or by selectively editing videos. Generative neural networks facilitate the creation of highly realistic deepfake videos that don’t require specialized expertise to create, opening a new front in the distribution of and fight against misinformation. Understanding vulnerabilities to deepfakes among key stakeholders and the factors that moderate susceptibility is critical for creating and implementing robust mitigation strategies. This study represents a first step at achieving those aims by analyzing a nationally representative set of U.S. adults, teachers, and principals, a large sample of middle school students throughout the country, and a sample of technically-oriented undergraduate and graduate students. Our results have three broad implications.

First, we found that deepfakes are already of sufficient quality as to introduce substantial confusion that leaves all education stakeholders vulnerable. Across all populations, between 27 percent to over a half of respondents were unable to correctly identify a video regardless of its authenticity. Across all videos, adult populations were more vulnerable than CMU and K-12 students, though all populations except for CMU students were less able to correctly identify the deepfake version of the video (compared to the authentic version) for at least two of four speakers. This gap between correctly identifying deepfake videos versus authentic videos was seen to be as large as 39 percentage points. These results have severe implications for the vulnerability of the U.S. education system. Vulnerabilities of K-12 students open the possibility that students take science misinformation as fact and develop a flawed view of science, the scientific process, and the policy implications of science. If these flawed views go unchallenged, they can solidify as students mature and enter positions in society where they can more directly influence policy. The more severe vulnerability of adults, and particularly of teachers and principals, is perhaps more alarming. Teachers and principals must synthesize and communicate contemporary issues in science to students and vulnerabilities to misinformation can translate to inadvertent teaching of information to students. Further, educators are even less equipped to debunk any misinformation students bring into the classroom as students increasingly become exposed to and rely on online information. The vulnerability of adults means that parents may not be able to debunk misinformation their children are exposed to and may play a role in spreading it.

Second, we found that focusing on technical aspects such as overall video quality or facial features can help respondents more accurately detect deepfakes but analyzing the social aspects such as knowledge of the speaker or the credibility of the content can help more accurately identify both deepfake *and* authentic videos. Thus, while prior studies focused on the more technical aspects of videos and the interplay between human and algorithmic detections, our results show that the social context of videos is also critical to consider. Educating information consumers to assess social characteristics of digital content may therefore present new focal points for strategies to mitigate vulnerabilities to deepfakes that may be more robust than a focus on technical aspects. Over the long term, deepfake generation technologies are expected to continue their rapid advancement towards producing content that is indistinguishable from authentic videos, eroding the utility of visually assessing technical aspects of deepfake videos.

Finally, we found several personal-level characteristics of individuals can guide the development and implementation of mitigation strategies. First, older individuals tended to exhibit higher levels of vulnerability and specific educational interventions should be tailored for this population. Second, higher trust in information sources, including in print, peer, and online sources, was found to raise respondents’ vulnerability to deepfakes, indicating that traditional media literacy approaches may also be effective in combatting deepfakes. Our results also suggest that other types of mitigation strategies, such as those that rely on repeated exposure, are unlikely to succeed and may worsen vulnerabilities. These latter results further highlight the need for education on deepfake detection, as repeated exposure to potential deepfakes can increase confusion and allow deepfakes to become a more potent medium by which to spread misinformation.

## Limitations

Though this study advances our knowledge of education stakeholders’ societal vulnerabilities to deepfake videos, including several nationally representative samples, this study also has some important limitations. First, the characteristics of videos between speakers were not varied in systematic ways, preventing us from understanding the discrete characteristics of deepfake videos that drive the heterogeneity in responses and vulnerabilities observed. Further research is needed to understand how specific features of deepfakes drive individuals’ vulnerability. Second, self-reported information on sensitive questions such as climate change, political ideology, and deepfakes can suffer from social desirability bias and may not always reflect the true view of the respondent. Though randomization meant that our main vulnerability results were not affected by this bias, the relationships between deepfake vulnerability and these aspects may have been affected. Third, the analysis of the relationships between video aspects and the probability of respondents correctly identifying deepfake and authentic videos are the only correlational analyses in this study. Though these results point to promising avenues for deepfake mitigation, more research is needed to establish causal connections. Finally, this study analyzed the specific type of misinformation where people’s views on climate change were manipulated. We chose our speakers to ensure variation in an array of salient characteristics including gender, age, professional positions, credentials, and notoriety. Nevertheless, we do not capture the full array of climate change speakers (e.g., politician) nor do they represent other types of possible deepfakes on climate change (e.g., misleading or fabricated scientific results). The results may also not generalize to misinformation on other topics such as vaccinations and it may not generalize to deepfakes that manipulate content in other ways.

## Methods

### Additional survey details

The survey consisted of two parts. In one part, respondents were asked to watch four videos. After each video, respondents were asked about the authenticity of the video and which aspects of the video helped them make their decision. The respondent could have identified the video as “Definitely Fake,” “Probably Fake,” “Cannot Tell,” “Probably Real,” or “Definitely Real.” These options allowed respondents to express a view on the authenticity of the video while providing an option (cannot tell) that suggests confusion in the respondent. Options regarding the aspects of the video that helped respondents make their decision include technical aspects (quality of the video, facial features of the speaker, background, and audio) as well as social aspects (familiarity of the respondent with the speaker’s view and credibility of the content of the video). These characteristics were chosen based on the literature on the creation of deepfakes and the social science literature on misinformation^20,55^.

The second part of the survey elicited the views of the respondents on a variety of dimensions. The constructs were chosen based on research that showed a relationship between these constructs and vulnerability to misinformation^12,21,55,56,59,66^. Where possible, we used or modified questions and scales that have been used in previous misinformation studies. Where no questions could be found, we created our own items. Appendix B contains the survey instrument and lists the sources of questions used from previous studies.

Theoretically, viewing the climate change associated videos prior to answering questions eliciting views on climate and deepfake videos could affect a respondent’s answer on those questions and vice versa. To guard against this possibility, we randomized whether the individual received the video or contextual questions first.

### Deepfake creation

The AI generated deepfake videos used in the survey were created via video-to-video synthesis^69^, a technique that generates artificial video content by transforming another video. The backbone of our video-to-video synthesis tool was the First Order Motion (FOM) model^25^, a machine learning model that takes two inputs, a driving video and a reference image, and yields a synthesized video that is an animation of the reference image according to the motion of objects in the driving video. To produce deepfakes related to climate change, we selected driving videos of individuals speaking in front of a camera about climate change and reference images of distinct individuals speaking in front of a camera. The audio component of the driving video was then added to the synthesized video. Following this procedure, we created four deepfake videos for our survey.

### Study populations

This survey leveraged three standing, nationally representative panels at the RAND Corporation: (1) The American Life Panel (ALP), a sample of adults 18 years of age or older; (2) the American School Leader Panel (ASLP), a sample of U.S. K-12 public school principals; and (3) American Teacher Panel (ATP), a sample of U.S. K-12 public school teachers. The ALP is weighted to be nationally representative on distributions of characteristics from the Current Population Survey Annual Social and Economic Supplement^70^. The ASLP and ATP are weighted based on distributions of characteristics from the National Center for Education Statistics’ Common Core of Data^71^. All three panels leverage probabilistic sampling and account for non-random non-response. The study sample included 761 unique individuals from the ALP, 740 from the ASLP, and 642 from the ATP.

The Challenger Center, a non-profit organization that provides experiential STEM education programs to students throughout the United States, recruited a sample of 805 middle school students in grades six through eight. Three Challenger Learning Centers recruited students from Kentucky, Maine, and Missouri. CMU recruited 87 graduate and undergraduate students from across the university. As these two samples were convenience samples, no weights were assigned to respondents.

In total, the analytical sample contained 3,035 respondents and 12,140 respondent-video observations. Table 4 provides the descriptive statistics for each population.

**Table 4:** Descriptive statistics.

	Adults			Principals			Teachers			Middle school students			CMU students		
	Mean	St. Err	N	Mean	St. Err	N	Mean	St. Err	N	Mean	St. Err	N	Mean	St. Err	N
Male	0.481	0.029	761	0.486	0.019	735	0.234	0.017	641	0.447	0.018	805	0.494	0.054	87
Age	52.614	0.898	761	49.179	0.299	739	44.172	0.425	642	12.653	0.038	792	20.276	0.162	87
White	0.756	0.026	761	0.643	0.018	740	0.684	0.019	642	0.862	0.012	805	0.345	0.051	87
Black	0.117	0.02	761	0.092	0.011	740	0.065	0.01	642	0.073	0.009	805	0.046	0.023	87
Hispanic	0.165	0.021	761	0.07	0.01	740	0.23	0.017	642	0.042	0.007	805	0.08	0.029	87
Other	0.127	0.021	761	0.098	0.011	740	0.09	0.012	642	0.065	0.009	805	0.609	0.053	87
Did not state race	0	0	761	0.167	0.014	740	0.161	0.015	642	0	0	805	0	0	87
HS or less	0.365	0.032	761	−	−	−	−	−	−	−	−	−	0.092	0.031	87
Some college	0.138	0.014	761	−	−	−	−	−	−	−	−	−	0.621	0.052	87
Employed	0.638	0.026	761	−	−	−	−	−	−	−	−	−	−	−	−
Married	0.644	0.028	761	−	−	−	−	−	−	−	−	−	−	−	−
Science occupation	0.123	0.016	761	−	−	−	−	−	−	−	−	−	−	−	−
Bachelor's degree	0.121	0.016	761	0.01	0.004	740	0.347	0.02	642	−	−	−	0.287	0.049	87
Masters or more	0.174	0.017	761	0.988	0.004	740	0.653	0.02	642	−	−	−	−	−	−
Born in US	0.884	0.019	761	−	−	−	−	−	−	−	−	−	0.724	0.048	87
Educator variables															
Years of experience	−	−	−	11.224	0.268	737	16.182	0.348	642	−	−	−	−	−	−
Title I eligible school	−	−	−	0.730	0.017	708	0.672	0.019	632	−	−	−	−	−	−
Elementary school	−	−	−	0.487	0.019	711	0.477	0.021	633	−	−	−	−	−	−
Middle school	−	−	−	0.229	0.016	711	0.207	0.016	633	−	−	−	−	−	−
High school	−	−	−	0.285	0.018	711	0.315	0.018	633	−	−	−	−	−	−
Math/science teacher	−	−	−	−	−	−	0.193	0.016	642	−	−	−	−	−	−
Social science teacher	−	−	−	−	−	−	0.068	0.01	642	−	−	−	−	−	−
Other teacher	−	−	−	−	−	−	0.739	0.017	642	−	−	−	−	−	−

Statistics from the adult, principal, and teacher populations are weighted to be nationally representative.

### Identification strategy and analysis

Randomization of deepfake videos to respondents means that the background characteristics and beliefs of respondents were uncorrelated with the receipt of authentic or deepfake videos. Table A7 in the Appendix presents tests of baseline balance of receipt of the deepfake videos on respondent and video characteristics. Of 170 statistical tests, 15 covariates were statistically significantly imbalanced to the 10 percent level and seven to the five percent level; a rate of imbalance that is expected by chance.

We analyzed the data at the respondent-video level to obtain an overall estimate of the vulnerability of populations to STEM deepfakes and answer research question 1. We leveraged models of the following form separately on each population:

1 $${Y}_{isr}= {\beta }_{0}+{\beta }_{1}{Deepfake}_{isr}+{\alpha }_{i}+{\gamma }_{s}+ {\tau }_{r}+{\varepsilon }_{isr}$$

where *Y*_*isr*_ was respondent, *i’s*, response to the video of speaker, *s*, randomized to be shown in the *rth* position (first video shown, second video shown, etc.)*.* We analyzed three outcomes of interest: correctly identifying the authenticity of the video, incorrectly doing so, and responding “Cannot Tell.” The respondent was coded as correct if they selected “Definitely Fake” or “Probably Fake” when receiving a deepfake video, or “Definitely Real” or “Probably Real” when receiving an authentic video. *Deepfake*_*isr*_ was an indicator for whether the video is a deepfake video, while *a*_*i*_, *g*_*s*,_ and *t*_*r*_ were respondent, speaker, and order fixed effects, respectively. No fixed effects were needed for identification, but were included to increase the precision of estimates. Finally, *e*_*isr*_ was a stochastic individual-level error term. Regressions using the ALP, ASLP, and ATP were weighted to retain national representativeness and all models used clustered standard errors at the respondent level to account for the correlation between each respondent’s response to the videos. We used linear probability models to ease the interpretation of the results and analyzed each population separately.

Recall that after each video we asked the respondents to indicate which of six video aspects helped them decide the video’s authenticity. To answer research question 2, we first investigated whether viewing a deepfake caused respondents to differentially use aspects of the video in their decision-making process. We leveraged models of the following form:

2 $${A}_{isr}= {\beta }_{0}+{\beta }_{1}{Deepfake}_{isr}+{\alpha }_{i}+{\gamma }_{s}+ {\tau }_{r}+{\varepsilon }_{isr}$$

Equation (2) is identical to Eq. (1) except *A*_*isr*_ was now one of the six video aspects. The coefficient of interest is *β*_*1*_, which indicated the extent to which respondents noted a particular video aspect was used to make their decision when receiving a deepfake. If *β*_*1*_ is small in magnitude and statistically insignificant, respondents are equally likely to have considered that video aspect in their decision-making progress, regardless of the authenticity of the video they are viewing. If *β*_*1*_ is positive (negative) and statistically significant, respondents are more (less) likely to have considered that aspect of a video when receiving a deepfake video.

We then investigate whether considering a video aspect leads to a higher likelihood of correctly detecting the video’s authenticity. We leveraged models of the following form:

3 $${Y}_{isr}= {\beta }_{0}+{{\varvec{A}}}_{{\varvec{i}}{\varvec{s}}{\varvec{r}}}{{\varvec{\beta}}}_{1}+{\alpha }_{i}+{\gamma }_{s}+ {\tau }_{r}+{\varepsilon }_{isr}$$

Equation (3) is the same as Eq. (1) except the indicator for obtaining a deepfake was replaced with a vector of the six video aspects, *A*_*isr*_. We estimated results separately for the subsamples of authentic and deepfake videos. The vector of coefficients of interest is ***β***_***1***_, which estimated the relationship between analyzing each video aspect and the probability of correctly, incorrectly, or being unable to identify the video’s authenticity. Because video aspects were not randomly assigned, these results were correlational.

Finally, we explore which subsets of populations are more vulnerable to deepfake videos to answer research question 3. We leveraged models of the following form:

4 $${Y}_{isr}= {\beta }_{0}+{\beta }_{1}{Deepfake}_{isr}+{\beta }_{2}{Deepfake}_{isr}*{X}_{i}+{\alpha }_{i}+{\gamma }_{s}+ {\tau }_{r}+{\varepsilon }_{isr}$$

Equation (4) was identical to Eq. (1) except we included an interaction term of the indicator for viewing the deepfake with the respondent characteristic of interest, *X*_*i*_. The coefficient of interest is *b*_*2*_, which provides the differential effect of receiving the deepfake video for the subgroup of interest compared to the reference group. We test the constructs previously detailed in the *Survey Instrument* section and the effect of the order of seeing the video. The larger number of individual and video characteristics could have presented a multiple hypothesis comparison problem. We did not formally correct for multiple hypotheses due to the exploratory nature of the analyses, but we guarded against it by only presenting results that showed a pattern of statistical significance across populations. In addition, we explored results from models that removed respondent fixed effects and include a vector of covariates of the characteristics in Table 4. We present results from both models to show stability of point estimates and robustness of statistical significance.

### Ethical considerations

The potential potency of deepfakes in spreading misinformation and the inclusion of vulnerable populations in the study, such as middle school children, raise important ethical considerations. On one hand these types of studies are essential because understanding how individuals react to deepfakes necessitate the controlled exposure to deepfakes. On the other hand, exposing individuals to deepfakes under any circumstances can have unintended effects. For example, our results indicate that repeated exposure to deepfakes can make individuals more susceptible to future deepfakes.

To balance the need for rigorous evidence with the safety of participants, the study team took several steps to minimize any harm the repeated exposure to potential deepfakes could cause. Prior to data collection, the study was approved by the Institutional Review Boards of RAND (registration ID: IORG0000034; studyID: 2020-N0613, Challenger Center (registration ID: IORG0001354; study ID: 8164-LBBush, and CMU (registration ID: IORG0000352; study ID: 2020_00000247) and the study was performed in accordance with all relevant guidelines and regulations including the declaration of Helsinki. As part of the procedures that were approved of the IRB, all participants provided informed consent before taking the survey. All adults provided informed consent directly. Schools opted into the study after an explanation of the procedures and secured parental informed consent before fielding the survey to middle school students. Respondents could also decline to take the survey in its entirety, answer any question, or stop taking the survey at any time with no penalty. Most importantly, each respondent was made aware of the authenticity of each video at the end the of the survey. For adult populations, the final page of the survey contained a screenshot of each video the respondent was exposed to and a clear indication of whether each video was authentic or a deepfake. For middle school students, the Challenger Center prepared a mini-lesson for teachers that clearly indicated the authenticity of each video and structured a discussion around deepfakes. The study team thought this more robust debrief was needed for middle school students because children are considered a vulnerable population.

These precautions ensured that no respondent left the study with a misinformed view of each speaker’s climate change belief. Whether these strategies were effective in mitigating future vulnerabilities to deepfakes is unknown. However, one important contribution of the study was to highlight the need for dedicated research to understand effective educational mitigation strategies, which our results indicate should include the social context of deepfakes.

### Supplementary Information

Supplementary Information 1.

Supplementary Information 2.

Supplementary Information 3.

## Data Availability

All data generated or analyzed during this study are included in this published article (and its Supplementary Information files).
