# Supplementary material for: Deepfakes and scientific knowledge dissemination
Source: Sci Rep. 2023 Aug 18;13:13429. doi: 10.1038/s41598-023-39944-3 (PMC10439167; doi:10.1038/s41598-023-39944-3)
Supplement: Supplementary file 1 — Supplementary Information 1. [file 41598_2023_39944_MOESM1_ESM.docx]

***Deepfakes and Scientific Knowledge Dissemination***

***Supplementary Information Appendices***

Christopher Doss^1*^

Jared Mondschein^1^

Dule Shu^2^

Tal Wolfson^3^

Denise Kopecky^4^

Valerie A. Fitton-Kane^4^

Lance Bush^4*^

Conrad Tucker^2*^

^1^RAND Corporation

^2^Carnegie Mellon University

^3^Pardee RAND Graduate School

^4^Challenger Center

**Appendix A: Supplemental Figures and Tables**

**
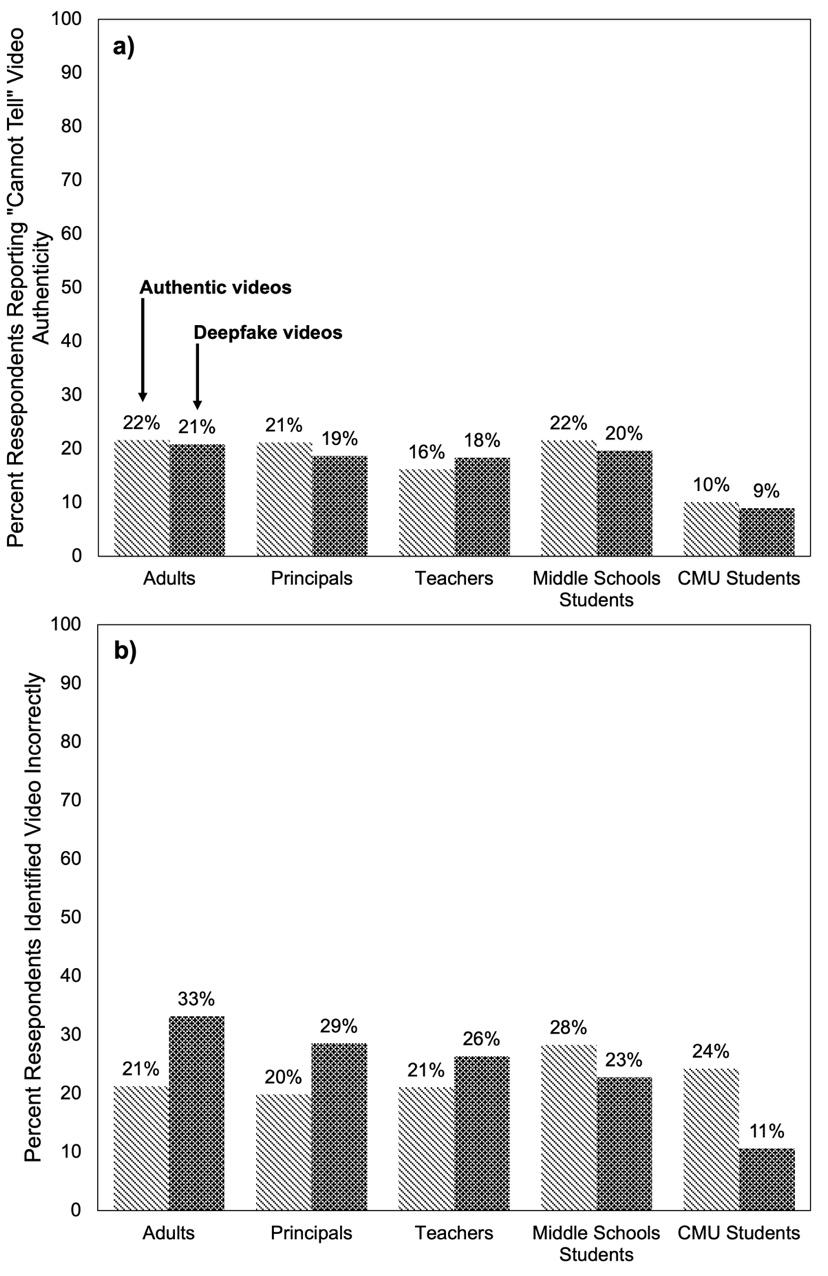
**

**Figure A1: Percent of Participants a) Responding Cannot Tell and b) Incorrectly Identifying the Authenticity of Videos by Video Authenticity and Population**

Notes: Each bar represents the percentage of responses that correctly identified the authenticity of videos by population and deepfake video status. Tabulations in the adult, principal, and teacher populations are weighted to be nationally representative.


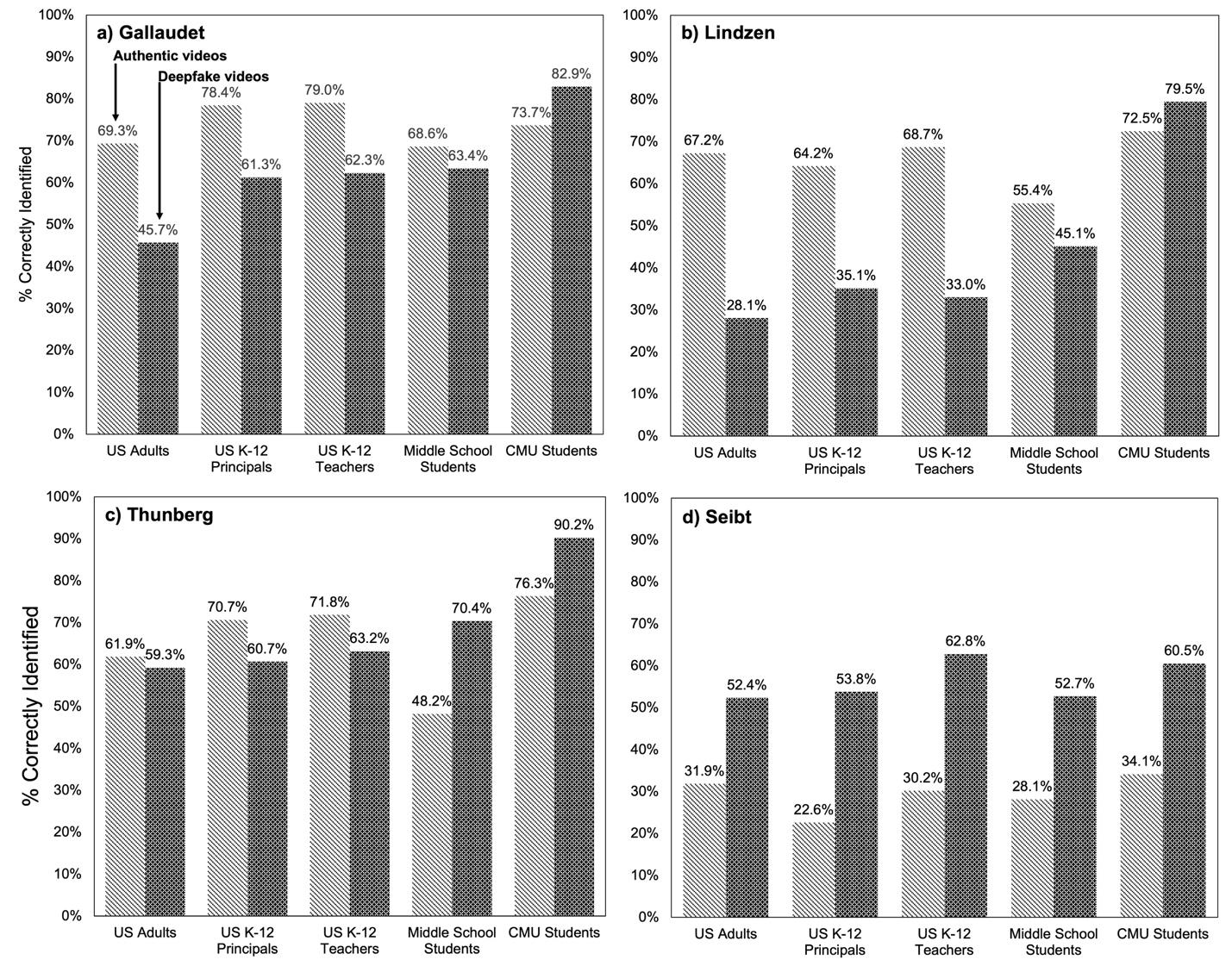


**Figure A2: Percent of Reponses Correctly Identifying the Authenticity of Videos, by Video Authenticity, Population, and Speaker**

Notes: Each bar represents the percentage of responses that correctly identified the authenticity of videos by population and deepfake video status. Tabulations in the adult, principal, and teacher populations are weighted to be nationally representative.


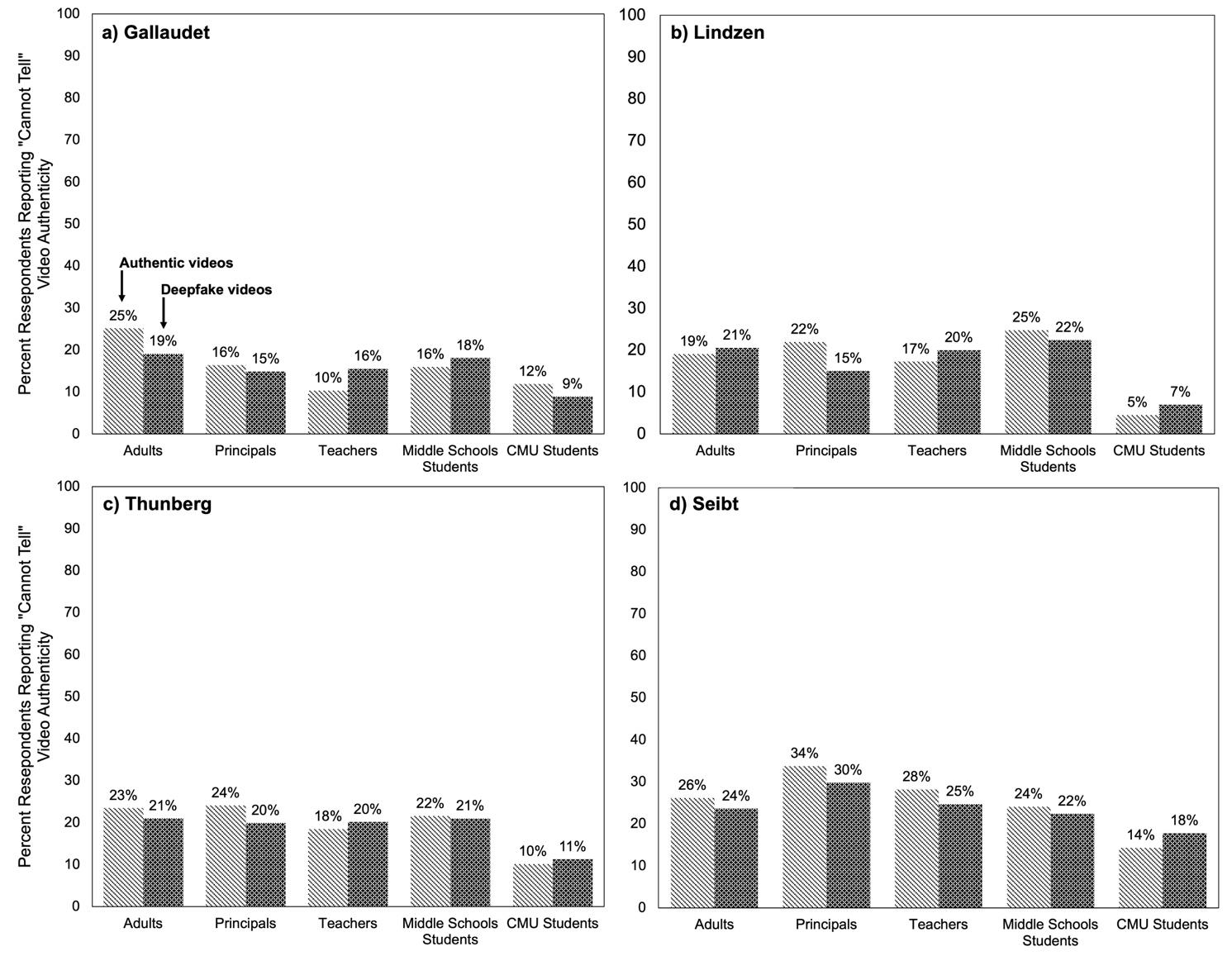


**Figure A3: Percent of Responses Reporting “Cannot Tell” Video Authenticity, by Video Authenticity, Population, and Speaker**

Notes: Each bar represents the percentage of responses that correctly identified the authenticity of videos by population and deepfake video status. Tabulations in the adult, principal, and teacher populations are weighted to be nationally representative.


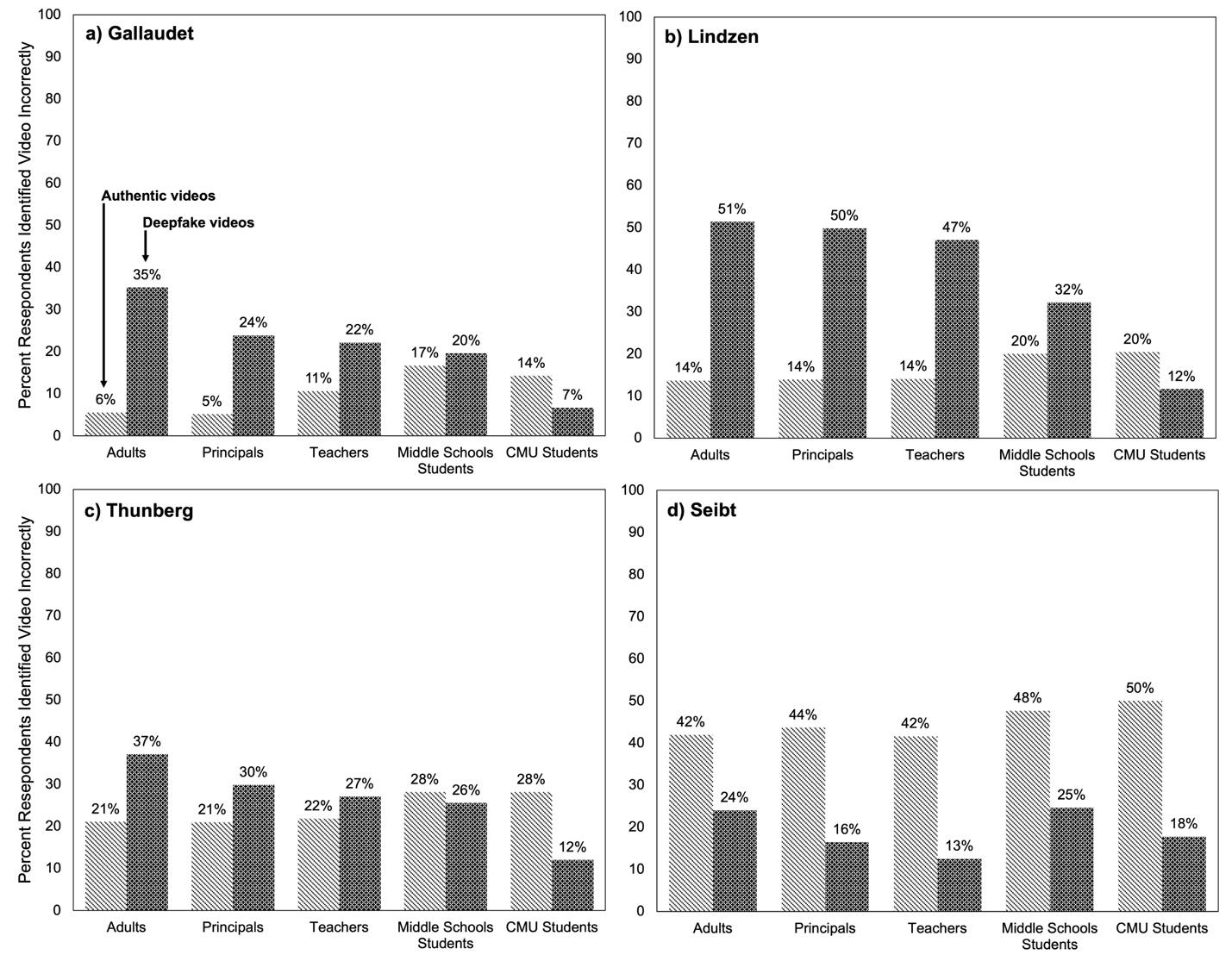


**Figure A4: Percent of Responses Identifying Video Incorrectly, by Video Authenticity, Population, and Speaker**

Notes: Each bar represents the percentage of responses that correctly identified the authenticity of videos by population and deepfake video status. Tabulations in the adult, principal, and teacher populations are weighted to be nationally representative.

Table A1: A Comparison of This Work to Relevant Prior Works

| Reference | Study Population | Population Size | Type of Deepfake | Focus of Deepfakes |
| --- | --- | --- | --- | --- |
| This study | Nationally representative samples of U.S. K-12 teachers, U.S. K-12 principals, and U.S. adults. Convenience samples of U.S. middle school students and AI/ML graduate students | 642 K-12 teachers; 740 K-12 principals; 761 U.S. adults; 805 middle school students; 87 graduate students | Videos | Climate change |
| Mai, 2023 | Convenience sample | 529 | Audio | Random |
| Lovato, 2023 | Convenience sample of U.S. adults | 2,016 | Videos | Random |
| Groh, 2022 | Convenience sample | 5,727 | Text, audio, videos | Politics |
| Groh, 2022 | Convenience sample | 15,016 | Videos | Random |
| Nightingale, 2022 | Convenience sample | 757 | Images | Faces |
| Ternovski, 2022 | Convenience sample | 1,396 | Videos | Politics |
| Yu, 2021 | Convenience sample of U.S., Polish, and Dutch adults | 2,413 U.S. adults; 2,164 Polish adults; 1,254 Dutch adults | Videos | Politics |
| Köbis, 2021 | Convenience sample of UK adults | 210 | Videos | Random |
| Vaccari, 2020 | Convenience sample | 2,005 | Videos | Politics |
| Dobber, 2020 | Convenience sample of Dutch adults | 278 | Videos, Audio | Politics |
| Shen, 2018 | Convenience sample of U.S. adults | 3,476 | Images | News articles |

| Table A2: Effect of Viewing a Deepfake Video on Responses Perceptions of Video Authenticity, All Speakers | | | | | | | | | | |
| --- | --- | --- | --- | --- | --- | --- | --- | --- | --- | --- |
|  | Adults | | Principals | | Teachers | | Middle School Students | | CMU Students | |
| Correct | -0.112** | -0.137** | -0.062** | -0.065** | -0.075** | -0.084** | 0.075** | 0.083** | 0.148** | 0.193** |
|  | (0.028) | (0.037) | (0.019) | (0.024) | (0.019) | (0.025) | (0.016) | (0.021) | (0.051) | (0.055) |
|  |  |  |  |  |  |  |  |  |  |  |
| Cannot Tell | -0.008 | 0.009 | -0.025+ | -0.017 | 0.022 | 0.016 | -0.019 | -0.031+ | -0.011 | -0.004 |
|  | (0.021) | (0.028) | (0.015) | (0.018) | (0.015) | (0.020) | (0.014) | (0.018) | (0.030) | (0.035) |
|  |  |  |  |  |  |  |  |  |  |  |
| Incorrect | 0.119** | 0.128** | 0.087** | 0.082** | 0.053** | 0.068** | -0.055** | -0.052** | -0.136** | -0.189** |
|  | (0.026) | (0.034) | (0.016) | (0.020) | (0.017) | (0.022) | (0.015) | (0.019) | (0.040) | (0.042) |
|  |  |  |  |  |  |  |  |  |  |  |
| Fixed Effects |  | **✓** |  | **✓** |  | **✓** |  | **✓** |  | **✓** |
|  |  |  |  |  |  |  |  |  |  |  |
| N(Respondent-by-Video) | 3044 | | 2,960 | | 2,568 | | 3,220 | | 348 | |
| N(Respondent) | 761 | | 740 | | 642 | | 805 | | 87 | |
| Notes: Each cell presents the results of a separate regression of an indicator for the respondent correctly identifying the authenticity of the video, incorrectly identifying the authenticity of the video, or stating that he or she cannot tell the authenticity of the video on an indicator for the video being a deepfake. Column headers indicate the population panels and row headers indicate the response. Unconditional models are presented as well as those with respondent fixed effects, speaker fixed effects, and order of presentation fixed effects. Regressions on adult, principal, and teacher samples are weighted to retain national representativeness. Standard errors are clustered by respondent. + indicates p < 0.10; ** p < 0.01 | | | | | | | | | | |

| Table A3: Effect of Deepfake Videos on Perceptions of Video Authenticity, By Speaker | | | | |
| --- | --- | --- | --- | --- |
| Panel A: Adults |  | | | |
|  | Gallaudet | Lindzen | Thunberg | Seibt |
| Correct | -0.230** | -0.403** | -0.019 | 0.252** |
|  | (0.048) | (0.047) | (0.050) | (0.047) |
| Cannot Tell | -0.040 | 0.026 | 0.028 | -0.047 |
|  | (0.043) | (0.040) | (0.039) | (0.040) |
| Incorrect | 0.270** | 0.377** | -0.009 | -0.205** |
|  | (0.035) | (0.045) | (0.041) | (0.047) |
| N(Respondent) | 761 | | | |
| Panel B: Principals |  | | | |
|  | Gallaudet | Lindzen | Thunberg | Seibt |
| Correct | -0.190** | -0.282** | -0.085* | 0.311** |
|  | (0.033) | (0.036) | (0.035) | (0.035) |
| Cannot Tell | -0.003 | -0.076* | 0.017 | -0.034 |
|  | (0.027) | (0.030) | (0.027) | (0.035) |
| Incorrect | 0.193** | 0.358** | 0.069* | -0.277** |
|  | (0.026) | (0.033) | (0.031) | (0.033) |
| N(Respondent) | 740 | | | |
| Panel C: Teachers |  | | | |
|  | Gallaudet | Lindzen | Thunberg | Seibt |
| Correct | -0.173** | -0.360** | -0.074* | 0.337** |
|  | (0.037) | (0.039) | (0.037) | (0.038) |
| Cannot Tell | 0.053+ | 0.037 | 0.020 | -0.032 |
|  | (0.028) | (0.033) | (0.027) | (0.035) |
| Incorrect | 0.120** | 0.324** | 0.054 | -0.304** |
|  | (0.030) | (0.036) | (0.034) | (0.034) |
| N(Respondent) | 642 | | | |
| Panel D: Middle School Students | | | | |
|  | Gallaudet | Lindzen | Thunberg | Seibt |
| Correct | -0.051 | -0.105** | 0.202** | 0.249** |
|  | (0.033) | (0.035) | (0.034) | (0.034) |
| Cannot Tell | 0.026 | -0.021 | -0.055* | -0.013 |
|  | (0.026) | (0.029) | (0.027) | (0.030) |
| Incorrect | 0.025 | 0.126** | -0.147** | -0.236** |
|  | (0.027) | (0.031) | (0.028) | (0.033) |
| N(Respondent) | 805 | | | |
| Panel D: CMU Students | | | | |
|  | Gallaudet | Lindzen | Thunberg | Seibt |
| Correct | 0.149 | 0.184+ | 0.067 | 0.238+ |
|  | (0.107) | (0.105) | (0.077) | (0.123) |
| Cannot Tell | -0.078 | -0.052 | -0.019 | 0.044 |
|  | (0.091) | (0.070) | (0.052) | (0.094) |
| Incorrect | -0.071 | -0.132 | -0.048 | -0.282* |
|  | (0.069) | (0.098) | (0.069) | (0.107) |
| N(Respondent) | 87 | | | |
| Notes: Each cell presents the results of a separate regression of an indicator for the respondent correctly identifying the authenticity of the video, incorrectly identifying the authenticity of the video, or stating that he or she cannot tell the authenticity of the video on an indicator for the video being a deepfake. Panel headers indicate the population, column headers indicate the speaker video, and row headers indicate the response. Population specific covariates included are listed in Table 4 of the main manuscript. CMU and middle school student models also contain state fixed effects. Regressions on adult, principal, and teacher samples are weighted to retain national representativeness. Robust standard errors in parentheses. + indicates p < 0.10; ** p < 0.01 | | | | |

| Table A4: Relationship Between Reported Aspect of Video Analyzed and Responding "I Can't Tell" Video Authenticity, By Video Deepfake Status | | | | | | | | | | |  |
| --- | --- | --- | --- | --- | --- | --- | --- | --- | --- | --- | --- |
|  | Adults | | Principals | | Teachers | | Middle School Students | | CMU Students | | |
|  | Real | Deepfake | Real | Deepfake | Real | Deepfake | Real | Deepfake | Real | Deepfake | |
| Familiar With Person's Views | -0.245** | -0.277* | -0.228** | -0.175** | -0.142* | -0.090 | -0.198** | -0.090 | -0.240+ | -0.051 | |
|  | (0.083) | (0.108) | (0.065) | (0.067) | (0.063) | (0.067) | (0.060) | (0.067) | (0.128) | (0.147) | |
| Video Quality | -0.076 | -0.078 | -0.052 | -0.035 | -0.021 | 0.029 | -0.023 | -0.045 | 0.080 | 0.085 | |
|  | (0.068) | (0.086) | (0.044) | (0.048) | (0.047) | (0.044) | (0.044) | (0.041) | (0.104) | (0.088) | |
| Background | 0.089 | -0.022 | 0.025 | -0.022 | 0.010 | 0.051 | -0.060 | -0.056 | 0.081 | -0.010 | |
|  | (0.130) | (0.078) | (0.050) | (0.056) | (0.049) | (0.059) | (0.043) | (0.043) | (0.104) | (0.102) | |
| Facial Features | -0.131+ | -0.126* | -0.083+ | -0.128** | -0.027 | -0.182** | -0.123* | -0.168** | -0.288* | -0.026 | |
|  | (0.072) | (0.064) | (0.048) | (0.045) | (0.047) | (0.052) | (0.048) | (0.042) | (0.131) | (0.142) | |
| Audio | -0.103 | -0.067 | -0.022 | -0.058 | -0.020 | -0.004 | -0.044 | -0.027 | -0.050 | 0.087 | |
|  | (0.071) | (0.068) | (0.050) | (0.048) | (0.045) | (0.045) | (0.048) | (0.045) | (0.114) | (0.110) | |
| Credibility of Content | -0.151* | -0.158* | -0.155** | -0.123** | 0.012 | -0.080 | -0.080 | -0.091+ | -0.213 | 0.021 | |
|  | (0.062) | (0.061) | (0.046) | (0.046) | (0.044) | (0.050) | (0.051) | (0.053) | (0.128) | (0.122) | |
|  |  |  |  |  |  |  |  |  |  |  | |
| N(Respondent-by-Video) | 1,600 | 1,444 | 1,514 | 1,446 | 1,283 | 1,285 | 1,600 | 1,620 | 169 | 179 | |
| N(Respondent) | 727 | 693 | 692 | 685 | 604 | 596 | 757 | 755 | 82 | 84 | |
|  |  |  |  |  |  |  |  |  |  |  | |
| Notes: Each column presents the results of a separate regression of video authenticity on aspects respondents reported analyzing when making their decisions. All models include speaker, video order, and respondent fixed effects. Regressions on adult, principal, and teacher samples are weighted to retain national representativeness. Standard errors are clustered by respondent. + indicates p < 0.10; * p < 0.05; ** p < 0.01. | | | | | | | | | | |  |

| Table A5: Relationship Between Reported Aspect of Video Analyzed and Responding Incorrectly, By Video Deepfake Status | | | | | | | | | | |  |
| --- | --- | --- | --- | --- | --- | --- | --- | --- | --- | --- | --- |
|  | Adults | | Principals | | Teachers | | Middle School Students | | CMU Students | | |
|  | Real | Deepfake | Real | Deepfake | Real | Deepfake | Real | Deepfake | Real | Deepfake | |
| Familiar With Person's Views | -0.136+ | 0.060 | -0.251** | -0.049 | -0.246** | -0.011 | -0.211** | 0.226** | -0.215 | 0.007 | |
|  | (0.080) | (0.104) | (0.061) | (0.075) | (0.066) | (0.071) | (0.064) | (0.076) | (0.229) | (0.220) | |
| Video Quality | 0.205* | -0.173* | 0.114* | -0.227** | 0.155** | -0.239** | 0.003 | -0.040 | -0.021 | 0.039 | |
|  | (0.081) | (0.076) | (0.048) | (0.051) | (0.059) | (0.048) | (0.048) | (0.047) | (0.160) | (0.096) | |
| Background | 0.046 | -0.066 | 0.050 | -0.061 | -0.020 | -0.028 | 0.057 | -0.070 | 0.054 | 0.085 | |
|  | (0.065) | (0.077) | (0.048) | (0.059) | (0.058) | (0.059) | (0.047) | (0.049) | (0.152) | (0.133) | |
| Facial Features | 0.164* | -0.026 | 0.020 | -0.026 | 0.017 | 0.049 | 0.033 | -0.029 | 0.299+ | 0.180 | |
|  | (0.079) | (0.070) | (0.044) | (0.052) | (0.054) | (0.052) | (0.050) | (0.047) | (0.158) | (0.145) | |
| Audio | -0.120+ | 0.068 | -0.200** | 0.144* | -0.220** | 0.105+ | -0.089+ | 0.136** | -0.101 | -0.013 | |
|  | (0.064) | (0.077) | (0.046) | (0.058) | (0.054) | (0.059) | (0.053) | (0.046) | (0.202) | (0.101) | |
| Credibility of Content | -0.006 | 0.201** | -0.024 | 0.196** | -0.076 | 0.171** | -0.014 | 0.175** | -0.001 | 0.044 | |
|  | (0.052) | (0.066) | (0.043) | (0.051) | (0.049) | (0.049) | (0.057) | (0.060) | (0.132) | (0.114) | |
|  |  |  |  |  |  |  |  |  |  |  | |
| N(Respondent-by-Video) | 1,600 | 1,444 | 1,514 | 1,446 | 1,283 | 1,285 | 1,600 | 1,620 | 169 | 179 | |
| N(Respondent) | 727 | 693 | 692 | 685 | 604 | 596 | 757 | 755 | 82 | 84 | |
|  |  |  |  |  |  |  |  |  |  |  | |
| Notes: Each column presents the results of a separate regression of video authenticity on aspects respondents reported analyzing when making their decisions. All models include speaker, video order, and respondent fixed effects. Regressions on adult, principal, and teacher samples are weighted to retain national representativeness. Standard errors are clustered by respondent. + indicates p < 0.10; * p < 0.05; ** p < 0.01. | | | | | | | | | | |  |

| Table A6: Additional Moderation Effects of Participant Background Characteristics on Correctly Identifying Deepfake Video | | | | | | | | | | | | | | | |  |  |
| --- | --- | --- | --- | --- | --- | --- | --- | --- | --- | --- | --- | --- | --- | --- | --- | --- | --- |
|  | Adults | | | Principals | | Teachers | | Middle School | | | | CMU | | |  |  |  |
| Male | -0.041 | -0.038 | | -0.008 | 0.007 | 0.022 | 0.038 | 0.052 | | 0.047 | | 0.148 | 0.099 | | | |  |
|  | (0.074) | (0.056) | | (0.048) | (0.037) | (0.058) | (0.045) | (0.042) | | (0.032) | | (0.110) | (0.099) | | | |  |
| Hispanic | 0.268* | 0.187* | | 0.004 | 0.014 | -0.004 | -0.021 | 0.041 | | 0.073 | | -0.211 | -0.384+ | | | |  |
|  | (0.104) | (0.078) | | (0.098) | (0.074) | (0.058) | (0.045) | (0.096) | | (0.071) | | (0.230) | (0.209) | | | |  |
| Black | 0.085 | -0.031 | | 0.094 | 0.122* | -0.099 | -0.055 | 0.093 | | 0.017 | | 0.192 | 0.467* | | | |  |
|  | (0.126) | (0.106) | | (0.083) | (0.061) | (0.088) | (0.072) | (0.095) | | (0.066) | | (0.378) | (0.214) | | | |  |
| Personal View of Global  Warming | -0.025 | -0.002 | | -0.096 | -0.085 | 0.039 | 0.030 | -0.055 | | -0.070+ | | 0.298** | 0.218* | | | |  |
|  | (0.078) | (0.060) | | (0.069) | (0.053) | (0.067) | (0.051) | (0.050) | | (0.037) | | (0.085) | (0.091) | | | |  |
| Perception of Scientific  View of Global Warming | -0.048 | -0.032 | | 0.022 | 0.050 | -0.024 | -0.019 | -0.035 | | -0.001 | | 0.101 | -0.103 | | | |  |
|  | (0.072) | (0.054) | | (0.055) | (0.042) | (0.054) | (0.040) | (0.042) | | (0.032) | | (0.141) | (0.107) | | | |  |
| Perceived Deepfake  Prevalence | 0.012 | 0.008 | | 0.015 | 0.021 | 0.052* | 0.045* | -0.007 | | -0.025 | | 0.017 | 0.014 | | | |  |
|  | (0.036) | (0.029) | | (0.025) | (0.019) | (0.024) | (0.019) | (0.022) | | (0.017) | | (0.054) | (0.047) | | | |  |
| Perceived Ability to Detect  Deepfakes | 0.011 | -0.022 | | -0.013 | -0.007 | 0.016 | 0.023 | 0.004 | | 0.005 | | -0.020 | -0.032 | | | |  |
|  | (0.037) | (0.028) | | (0.025) | (0.019) | (0.024) | (0.020) | (0.022) | | (0.017) | | (0.048) | (0.044) | | | |  |
| Perceived Risk of  Deepfakes to Self | -0.037 | -0.028 | | 0.007 | 0.013 | 0.019 | 0.013 | 0.055** | | 0.026 | | -0.062 | -0.068 | | | |  |
|  | (0.034) | (0.025) | | (0.024) | (0.019) | (0.025) | (0.019) | (0.021) | | (0.016) | | (0.052) | (0.045) | | | |  |
| Perceived Risk of  Deepfakes to Society | 0.025 | 0.009 | | 0.004 | 0.015 | 0.001 | 0.000 | 0.005 | | 0.001 | | -0.028 | -0.027 | | | |  |
|  | (0.030) | (0.024) | | (0.022) | (0.018) | (0.024) | (0.019) | (0.021) | | (0.016) | | (0.054) | (0.043) | | | |  |
| Total Climate Change Quiz  Questions Correct | -0.003 | -0.004 | | -0.012 | 0.004 | 0.015 | 0.011 | 0.006 | | 0.006 | | 0.053 | 0.054 | | | |  |
|  | (0.038) | (0.030) | | (0.029) | (0.022) | (0.031) | (0.023) | (0.022) | | (0.017) | | (0.079) | (0.072) | | | |  |
| Number of Internet Devices  Used | -0.008 | -0.014 | | -0.038 | -0.023 | 0.035 | 0.015 | -0.016 | | 0.009 | | -0.061 | -0.070 | | | |  |
|  | (0.067) | (0.049) | | (0.034) | (0.026) | (0.035) | (0.025) | (0.026) | | (0.020) | | (0.074) | (0.065) | | | |  |
| Factor of Climate Change  Learning Habits | 0.019 | 0.006 | | -0.017 | -0.024 | 0.026 | 0.019 | 0.006 | | 0.014 | | -0.060 | -0.078 | | | |  |
|  | (0.038) | (0.028) | | (0.025) | (0.019) | (0.026) | (0.022) | (0.026) | | (0.019) | | (0.067) | (0.051) | | | |  |
| Factor of Social Media  Platform Use | 0.155** | 0.122** | | -0.008 | -0.022 | -0.050 | -0.037 | -0.029 | | -0.029 | | -0.035 | -0.020 | | | |  |
|  | (0.051) | (0.041) | | (0.030) | (0.023) | (0.031) | (0.024) | (0.026) | | (0.020) | | (0.084) | (0.071) | | | |  |
| U.S. Born | -0.167 | -0.094 | | --- | --- | --- | --- | --- | | --- | | 0.168 | 0.065 | | | |  |
|  | (0.140) | (0.108) | | --- | --- | --- | --- | --- | | --- | | (0.120) | (0.116) | | | |  |
| Above Median U.S. Income | 0.017 | 0.008 | | --- | --- | --- | --- | --- | | --- | | --- | --- | | | |  |
|  | (0.074) | (0.055) | | --- | --- | --- | --- | --- | | --- | | --- | --- | | | |  |
| Live in an Urban Centric  Locale | 0.071 | 0.059 | | --- | --- | --- | --- | --- | | --- | | --- | --- | | | |  |
|  | (0.081) | (0.057) | | --- | --- | --- | --- | --- | | --- | | --- | --- | | | |  |
| Work In a Science Related  Field | 0.081 | 0.092 | | --- | --- | --- | --- | --- | | --- | | --- | --- | | | |  |
|  | (0.135) | (0.094) | | --- | --- | --- | --- | --- | | --- | | --- | --- | | | |  |
| Title I Eligible School | --- | --- | | -0.018 | 0.008 | -0.008 | -0.036 | --- | | --- | | --- | --- | | | |  |
|  | --- | --- | | (0.055) | (0.043) | (0.054) | (0.040) | --- | | --- | | --- | --- | | | |  |
| Years of Experience in  Educator Role | --- | --- | | -0.000 | 0.000 | -0.000 | -0.002 | --- | | --- | | --- | --- | | | |  |
|  | --- | --- | | (0.004) | (0.003) | (0.003) | (0.002) | --- | | --- | | --- | --- | | | |  |
| Works in Middle School  (vs. Elementary) | --- | --- | | -0.107 | -0.096+ | 0.007 | 0.003 | --- | | --- | | --- | --- | | | |  |
|  | --- | --- | | (0.066) | (0.051) | (0.069) | (0.052) | --- | | --- | | --- | --- | | | |  |
| Works in High School (vs.  Elementary) | --- | --- | | -0.074 | -0.020 | 0.040 | 0.025 | --- | | --- | | --- | --- | | | |  |
|  | --- | --- | | (0.069) | (0.055) | (0.059) | (0.044) | --- | | --- | | --- | --- | | | |  |
| Works in Other Type of  School (vs. Elementary) | --- | --- | | -0.080 | -0.041 | -0.066 | -0.080 | --- | | --- | | --- | --- | | | |  |
|  | --- | --- | | (0.060) | (0.046) | (0.091) | (0.075) | --- | | --- | | --- | --- | | | |  |
| Teach Social Studies  (vs. All Other Teachers Except Math/Science) | --- | --- | | --- | --- | 0.002 | 0.010 | --- | | --- | | --- | --- | | | |  |
|  | --- | --- | | --- | --- | (0.086) | (0.082) | --- | | --- | | --- | --- | | | |  |
| Teach Math/Science  (vs. All Other Teachers Except Social Studies) | --- | --- | | --- | --- | 0.035 | -0.012 | --- | | --- | | --- | --- | | | |  |
|  | --- | --- | | --- | --- | (0.045) | (0.046) | --- | | --- | | --- | --- | | | |  |
|  |  |  | |  |  |  |  |  | |  | |  |  | | | |  |
| Respondent Fixed Effects | **✓** |  | | **✓** |  | **✓** |  | **✓** | |  | | **✓** |  | | | |  |
| Respondent Controls |  | **✓** | |  | **✓** |  | **✓** |  | | **✓** | |  | **✓** | | | |  |
|  |  |  | |  |  |  |  |  | |  | |  |  | | | |  |
| N(Respondent-by-Video) | 3044 | | 2,960 | | | 2,568 | | | 3,220 | | 348 | | |  |  |  |  |
| N(Respondent) | 761 | | 740 | | | 642 | | | 805 | | 87 | | |  |  |  |  |
| Notes: Each cell presents the interaction term of a separate regression of whether a respondent correctly identified the authenticity of a video on the main effect for seeing a fake video and an interaction with the characteristic indicated by the row headers. All models include speaker and video order fixed effects. Models include respondent fixed effects or respondent controls as indicated in the table. Respondent controls are listed in Table 4 of the main manuscript. CMU and middle school student models additionally contain state fixed effects. Regressions on adult, principal, and teacher samples are weighted to retain national representativeness. Standard errors are clustered by respondent. + indicates p < 0.10; * p < 0.05; ** p < 0.01. | | | | | | | | | | | | | | | | | |

| Table A7: Relationship Between Receiving Deepfake and Respondent Characteristics, Covariate Balance Across All Speakers | | | | | |
| --- | --- | --- | --- | --- | --- |
|  | Adults | Principals | Teachers | Middle School Students | CMU Students |
| Gallaudet Video | 0.053 | 0.019 | -0.011 | -0.012 | 0.004 |
|  | (.033) | (.022) | (.024) | (.02) | (.066) |
| Lindzen Video | 0.012 | -0.009 | -0.001 | 0.008 | -0.027 |
|  | (.035) | (.021) | (.023) | (.02) | (.059) |
| Thunberg Video | -0.037 | -0.016 | -0.010 | 0.008 | 0.019 |
|  | (.034) | (.022) | (.024) | (.021) | (.068) |
| Seibt Video | -0.027 | 0.006 | 0.021 | -0.005 | 0.004 |
|  | (.035) | (.022) | (.023) | (.021) | (.059) |
| Male | .046+ | -0.026 | -0.009 | 0.028 | -0.051 |
|  | (.026) | (.019) | (.023) | (.017) | (.052) |
| Age in Years | 0.000 | 0.001 | -.002* | -0.005 | 0.015 |
|  | (.001) | (.001) | (.001) | (.008) | (.018) |
| Hispanic | -0.043 | -0.007 | -.049* | -0.011 | -.132+ |
|  | (.036) | (.038) | (.024) | (.039) | (.075) |
| Black | 0.075 | 0.045 | -0.011 | 0.001 | 0.181 |
|  | (.057) | (.036) | (.039) | (.04) | (.14) |
| Saw Video Before Questions | -0.025 | 0.021 | 0.018 | 0.014 | 0.035 |
|  | (.026) | (.019) | (.021) | (.017) | (.053) |
| Video Was First in Order | -0.037 | 0.012 | 0.004 | -0.025 | -0.027 |
|  | (.033) | (.022) | (.024) | (.021) | (.064) |
| Video Was Second in Order | 0.004 | 0.013 | -0.009 | -0.005 | 0.004 |
|  | (.038) | (.022) | (.024) | (.02) | (.061) |
| Video Was Third in Order | 0.027 | -0.010 | -0.011 | .048* | -0.011 |
|  | (.033) | (.022) | (.023) | (.02) | (.064) |
| Video Was Fourth in Order | 0.006 | -0.015 | 0.016 | -0.018 | 0.034 |
|  | (.032) | (.022) | (.023) | (.021) | (.064) |
| Understood Cause of Climate Change | -0.032 | -0.040 | -0.034 | -0.024 | 0.101 |
|  | (.03) | (.026) | (.026) | (.02) | (.074) |
| Thought Climate Change Was Man Made | -0.026 | -0.011 | 0.028 | -0.001 | 0.121 |
|  | (.027) | (.021) | (.022) | (.017) | (.118) |
| Perceived Prevalence of Deepfakes | 0.013 | -0.005 | -0.013 | 0.001 | -0.007 |
|  | (.012) | (.01) | (.01) | (.008) | (.023) |
| Perceived Ability to Detect Deepfakes | 0.022 | -0.006 | -0.001 | 0.013 | 0.022 |
|  | (.016) | (.01) | (.011) | (.008) | (.025) |
| Perceived Risk of Deepfakes to Self | 0.010 | -.019* | .018+ | -0.004 | 0.033 |
|  | (.013) | (.01) | (.011) | (.009) | (.022) |
| Perceived Risk of Deepfakes to Society | -0.001 | -.019* | -0.001 | -0.010 | 0.000 |
|  | (.011) | (.009) | (.011) | (.009) | (.028) |
| Total Climate Change Quiz Question Correct | 0.008 | -0.016 | -0.004 | -.020* | -0.014 |
|  | (.014) | (.013) | (.013) | (.009) | (.042) |
| Total Internet Devices at Home | 0.022 | 0.008 | -0.009 | 0.004 | 0.053 |
|  | (.016) | (.014) | (.015) | (.012) | (.036) |
| Factor of Learning Frequency | -0.005 | 0.000 | .019+ | -0.005 | -0.038 |
|  | (.015) | (.01) | (.011) | (.01) | (.03) |
| Factor of Trust in Information Sources | -.028* | -0.011 | -0.006 | -0.003 | -0.009 |
|  | (.014) | (.011) | (.012) | (.01) | (.025) |
| Factor of Use of Online Platforms | 0.008 | .020+ | 0.017 | -0.011 | -0.045 |
|  | (.013) | (.012) | (.013) | (.01) | (.037) |
| Identifies as Liberal | -0.017 | -0.027 | 0.002 | --- | -0.054 |
|  | (.024) | (.02) | (.022) | --- | (.054) |
| Identifies as Conservative | 0.026 | .035+ | -0.027 | --- | -0.015 |
|  | (.027) | (.021) | (.023) | --- | (.065) |
| Identifies as Moderate | -0.045 | -0.018 | 0.015 | --- | 0.097 |
|  | (.034) | (.021) | (.024) | --- | (.072) |
| Prefers Not to Disclose Political Orientation | 0.046 | 0.025 | 0.022 | --- | -0.016 |
|  | (.046) | (.033) | (.03) | --- | (.082) |
| Married | -0.019 | --- | --- | --- | --- |
|  | (.03) | --- | --- | --- | --- |
| Born in the US | -0.058 | --- | --- | --- | -0.009 |
|  | (.052) | --- | --- | --- | (.056) |
| Employed | -0.025 | --- | --- | --- | --- |
|  | (.028) | --- | --- | --- | --- |
| Has Medical Insurance | -0.058 | --- | --- | --- | --- |
|  | (.047) | --- | --- | --- | --- |
| Owns a House | -0.018 | --- | --- | --- | --- |
|  | (.028) | --- | --- | --- | --- |
| Urban Locale | 0.037 | --- | --- | --- | --- |
|  | (.03) | --- | --- | --- | --- |
| Some College | -0.007 | --- | --- | --- | -0.074 |
|  | (.029) | --- | --- | --- | (.056) |
| Bachelor's Degree | 0.023 | --- | -0.017 | --- | 0.050 |
|  | (.029) | --- | (.022) | --- | (.062) |
| Masters Degree or Higher | -0.011 | --- | 0.017 | --- | --- |
|  | (.029) | --- | (.022) | --- | --- |
| Income Below U.S. Median | -0.003 | --- | --- | --- | --- |
|  | (.026) | --- | --- | --- | --- |
| Science Related Occupation | 0.045 | --- | --- | --- | --- |
|  | (.034) | --- | --- | --- | --- |
| Title I Eligible School | --- | 0.002 | 0.028 | --- | --- |
|  | --- | (.022) | (.022) | --- | --- |
| Years of Experience |  | .003+ | -.002+ | --- | --- |
|  | --- | (.001) | (.001) | --- | --- |
| Elementary School Educator | --- | 0.026 | -0.014 | --- | --- |
|  | --- | (.021) | (.021) | --- | --- |
| Middle School Educator | --- | 0.000 | -0.032 | --- | --- |
|  | --- | (.026) | (.027) | --- | --- |
| High School Educator | --- | -0.019 | 0.029 | --- | --- |
|  | --- | (.022) | (.021) | --- | --- |
| Educator in Other Type of School | --- | -0.012 | 0.032 | --- | --- |
|  | --- | (.021) | (.041) | --- | --- |
| Math or Science Teacher | --- | --- | 0.010 | --- | --- |
|  | --- | --- | (.023) | --- | --- |
| Social Studies Teacher | --- | --- | -0.002 | --- | --- |
|  | --- | --- | (.038) | --- | --- |
| Teaches Other Subject | --- | --- | -0.007 | --- | --- |
|  | --- | --- | (.022) | --- | --- |
|  |  |  |  |  |  |
| N(Respondent-by-Video) | 3,032 - 3,044 | 2,832 - 2,960 | 2,528 - 2,568 | 2,756 - 3,220 | 316-348 |
| N(Respondent) | 761-758 | 708-740 | 632-642 | 806-689 | 79-87 |
| Notes: Each cell represents a separate regression of the respondent or video characteristic on an indicator for receiving a deepfake version of the video. Regressions on adult, principal, and teacher samples are weighted to retain national representativeness. Standard errors are clustered at the respondent level. + indicates p < 0.10; * p< 0.05; ** p< 0.01 | | | | | |

**References**

1. C. Shen, M. Kasra, W. Pan, G.A. Bassett, Y. Malloch, and J.F. O’Brien. Fake images: The effects of source, intermediary, and digital media literacy on contextual assessment of image credibility online. *New media & society* **21**, 2 (2019).
2. J. Ternovski, J. Kalla, and P.M. Aronow. The negative consequences of informing voters about deepfakes: Evidence from two survey experiments. *Journal of Online Trust & Safety* (2022).
3. C. Vaccari, and A. Chadwick. Deepfakes and disinformation: Exploring the impact of synthetic political video on deception, uncertainty, and trust in news. *Social Media + Society* (2020).
4. M. Groh, A. Sankaranarayanan, and R. Picard. Human detection of political deepfakes across transcripts, audio, and video. *ArXiv preprint*, arXiv: 2202.12883v1 (2022).
5. S.J. Nightingale, and H. Farid. AI-synthesized faces are indistinguishable from real faces and more trustworthy. *Proceedings of the National Academy of Science* **119**, 8 (2022).
6. J T. Dobber, N. Metoui, D. Trilling, N. Helberger, and C. de Vreese, Do (microtargeted) deepfakes have real effects on political attitudes? *The International Journal of Press/Politics* **26**, 1 (2020).
7. X. Yu, M. Wojcieszak, S. Lee, A. Casas, R. Azrout, and T. Gackowski. The (null) effects of happiness on affective polarization, conspiracy endorsement, and deep fake recognition: Evidence from five survey experiments in three countries. *Political Behavior* **43**, (2021).
8. J. Lovato, L. Hébert-Dufresne, J. St-Onge, R. Harp, G.S. Lopez, S.P. Rogers, I.U. Haq, and J. Onaolapo. Diverse misinformation: Impacts of human biases on detection of deepfakes on networks. *ArXiv preprint*, arXiv: 2210.10026v2 (2023).
9. N.C. Köbis, B. Doležalová, and I. Soraperra. Fooled twice: People cannot detect deepfakes but think they can. *iScience* **24**, (2021).
10. M. Groh, Z. Epstein, and R. Picard. Deepfake detection by human crowds, machines, and machine-informed crowds. *Proceedings of the National Academy of Science* **119**, 1 (2022).
11. K.T. Mai, S. Bray, T. Davies, and L.D. Griffin. Warning: Humans cannot reliably detect speech deepfakes. *ArXiv preprint*, arXiv: 2301.07829v1 (2023).

**Appendix B: Survey Instrument and Videos**

*Note: Numbers in brackets after a question indicate the study off which the question is based. See References for corresponding citation. If a question does not have a study referenced, that question was created by the study team.*

You will be shown a series of videos. For each video, please tell us how confident you are that the video is either real or fake. For this survey, “fake” means that one or more things in the video have been changed and “real” means that nothing in the video has been changed.

*1.* *Videos*

1a. How likely is this video fake or real? [1]

1. Definitely Fake

2. Probably Fake

3. Cannot Tell

4. Probably Real

5. Definitely Real

1b. Which aspects of the video below helped you decide if the video was real or fake? (Check all that apply) [2]

a. I am familiar with this person’s views

b. Video quality

c. Background

d. Facial feature(s) (e.g., mouth, eyes, nose, etc.)

e. Audio (sound)

f. Credibility of content

g. Other: (Please specify)

*2.* *Climate Change Beliefs*

2a. Which comes closer to your own view? [3]

a. Most scientists think global warming is happening

b. Most scientists think global warming is not happening

c. There is a lot of disagreement among scientists about whether or not global warming is

happening

d. I don’t know enough to say

2b. Which of the following best describes your view of global warming? [3]

a. Global warming is happening and is caused mostly by human activities

b. Global warming is happening and is caused by both human activities and natural

changes in the environment

c. Global warming is happening and is caused mostly by natural changes in the

environment

d. Global warming isn’t happening

e. I don’t know enough to say

f. Other (Please specify)

*3.* *Knowledge of Climate Science*

3a. The “greenhouse effect” refers to: (Choose the best option) [3]

a. Gases in the atmosphere that trap heat

b. The Earth’s protective ozone layer

c. Pollution that causes acid rain

d. How plants grow

e. Don’t know

3b. What gas is produced by the burning of fossil fuels? (Choose the best option) [3]

1. Oxygen
2. Hydrogen
3. Helium
4. Carbon dioxide
5. Don’t know

3c. Which of the following causes ocean acidification? (Choose the best option) [3]

a. Absorption of carbon dioxide by the ocean

b. Chemical spills in the ocean

c. Acid rain

d. Warmer ocean temperatures

e. Don’t know

*4.* *Political Affiliation (asked of everyone except middle school students)*

Which of the following best describes your political orientation? [4]

a. Very liberal

b. Liberal

c. Lean liberal

d. Moderate

e. Lean conservative

f. Conservative

g. Very conservative

h. Prefer not to say

i. Other

*5.* *Learning Habits*

5a. On average, how often do you learn about climate change from the following sources?

(Never, less than once a month, a couple times a month, weekly, daily, multiple times a day) [5]

a. Peers/colleagues

b. Teachers/Professors

c. Other adults

d. Social media (Facebook, Twitter, Instagram, Tik Tok, YouTube, blogs, etc.)

e. News sources (News Websites, News Programs, Newspapers, etc.)

f. Government agencies

g. Internet searches

h. Books or magazines

i. Other (please specify):

5b. How much do you trust the news and information about climate change that you learn from:

(Not at all, somewhat distrust, unsure, somewhat trust, absolutely trust, Not Applicable) [5]

a. Peers/colleagues

b. Teachers/Professors

c. Scientists

d. Other adults

e. Social media (Facebook, Twitter, Instagram, Tik Tok, YouTube, blogs, etc.)

f. News sources (News Websites, News Programs, Newspapers, etc.)

g. Government agencies

h. Internet searches

i. Books or magazines

j. Other (please specify):

*6.* *Internet Use Habits*

6a. During non-work hours, which device do you primarily use to access the internet? (check all that apply) [6]

a. Personal computer

b. Smartphone

c. Tablet

d. Public computer (e.g., at a library or community center)

e. No devices

f. Other (Please specify)

6b. How often do you use the following digital platforms for any reason? (Response Options: Never, Less Than Once a Month, A Couple Times a Month, Weekly, Daily, Multiple Times a Day) [7]

a. Facebook

b. Twitter

c. Instagram

d. Tik Tok

e. YouTube

f. Blogs

g. News Websites

h. Other: Please specify

6c. What device are you using to respond to this survey?

a. Computer

b. Smartphone

c. Tablet

*7.* *Perceptions of Deep Fakes*

7a. How commonly do you think fake science, fake technology, fake engineering, or fake math videos or images are seen on the Internet? [doesn’t exist, rare, somewhat rare, somewhat common, common, it’s everywhere]

7b. How much risk do you think fake science, fake technology, fake engineering, or fake math videos or images on the Internet pose to successfully completing your professional work? Response options: No risk, Slight risk, Moderate risk, Great risk, Not applicable

7c. How often do you think you are able to detect fake science, fake technology, fake engineering or fake math videos or images? Response Options: None of the time, Sometimes, Most of the time, All of the time

7d. How much risk does fake digital science, fake technology, fake engineering, or fake math videos or images pose to the overall functioning of society? Response options: No risk, Slight risk, Moderate risk, Great risk

*8.* *Background Questions*

| **Item** | **Population** |
| --- | --- |
| Gender: (Male/Female) | All Populations |
| Date of birth (age) | All Populations |
| Household Income | General Adults |
| Educational attainment (less HS, HS/GED, some college, BA, MA or above) | Teachers, Principals, General Adults, Carnegie Mellon University Students |
| Occupation | General Adults |
| Race (White, African-American/Black, Asian or Pacific Islander, American Indian/Alaska Native, Other) | All Populations |
| Hispanic/Latino (yes/no) | All Populations |
| Grade Level Taught | Teachers |
| School level (elementary, middle, high, K-8) | Teachers, Principals |
| Title I school status | Teachers, Principals |
| Subject Taught | Teachers |
| Years of Experience | Teachers, Principals |

**Video Links**

***Please not the links may open a full screen video on your browser. Respondents saw the videos in a smaller window.***

Timothy Gallaudet:

Fake: <https://cmu.ca1.qualtrics.com/CP/File.php?F=F_6G5PaVsaY4bk8hU>

Narrative: Video Timothy Gallaudet, Ph.D. in oceanography and former acting administrator (10.25.2017 - 02.28.2019) of the National Oceanic and Atmospheric Administration (NOAA), shares his opinion about climate change in the event of "Blue Tech Week".

Real: [https://cmu.ca1.qualtrics.com/CP/File.php?F=F_9ozlhu5tQz7X314](https://protect2.fireeye.com/v1/url?k=934a9fb9-ccd1a745-934ab1ee-ac1f6b733ff0-20ae26fbefc61600&q=1&e=1d7ba9fb-5776-4722-a283-a5c6ced696a8&u=https%3A%2F%2Fcmu.ca1.qualtrics.com%2FCP%2FFile.php%3FF%3DF_9ozlhu5tQz7X314)

Narrative: Timothy Gallaudet, Ph.D. in oceanography and former acting administrator (10.25.2017 - 02.28.2019) of the National Oceanic and Atmospheric Administration (NOAA). Prior to joining NOAA, Dr. Gallaudet explains why climate change matters to the Department of Defense (DOD) at the United States' 2017 Sea-Air-Space Exposition, while serving as the oceanographer and navigator of the Navy and commander of Naval Meteorology and Oceanography Command.

Richard Lindzen:

Fake: <https://cmu.ca1.qualtrics.com/CP/File.php?F=F_eCJtfm1KHMDWKge>

Narrative: Richard Lindzen, former professor at Massachusetts Institute of Technology (MIT), explains the potential threat of global warming in an interview with The Boston Globe.

Real: [https://cmu.ca1.qualtrics.com/CP/File.php?F=F_3OeSJDVRC6pKG8e](https://protect2.fireeye.com/v1/url?k=8652f4ad-d9c9cc51-8652dafa-ac1f6b733ff0-51af255d6ba856a8&q=1&e=1d7ba9fb-5776-4722-a283-a5c6ced696a8&u=https%3A%2F%2Fcmu.ca1.qualtrics.com%2FCP%2FFile.php%3FF%3DF_3OeSJDVRC6pKG8e)

Narrative: Richard Lindzen, former professor at Massachusetts Institute of Technology (MIT), explains why we shouldn't rush to a consensus about climate change from a scientific point of view in an interview with The Boston Globe.

Greta Thunberg:

Fake: [https://cmu.ca1.qualtrics.com/CP/File.php?F=F_3xadkhokipbqXZk](https://protect2.fireeye.com/v1/url?k=e584dcf4-ba1fe408-e584f2a3-ac1f6b733ff0-e041f328f39e8d4a&q=1&e=1d7ba9fb-5776-4722-a283-a5c6ced696a8&u=https%3A%2F%2Fcmu.ca1.qualtrics.com%2FCP%2FFile.php%3FF%3DF_3xadkhokipbqXZk)

Narrative: Greta Thunberg, a Swedish environmental activist who is internationally known for challenging world leaders to take immediate actions against climate change, alters her opinion on climate change when interviewed by a TV news channel.

Real: <https://cmu.ca1.qualtrics.com/CP/File.php?F=F_3VF7d0A7yy51EdU>

Narrative: Greta Thunberg, a Swedish environmental activist who is internationally known for challenging world leaders to take immediate actions against climate change, explains why we cannot wait to address the global warming crisis on an interview.

Naomi Seibt:

Fake: [https://cmu.ca1.qualtrics.com/CP/File.php?F=F_3yjWbA5H9yDVMpg](https://protect2.fireeye.com/v1/url?k=e46f41b2-bbf4794e-e46f6fe5-ac1f6b733ff0-84d02ddab1b7b9ae&q=1&e=1d7ba9fb-5776-4722-a283-a5c6ced696a8&u=https%3A%2F%2Fcmu.ca1.qualtrics.com%2FCP%2FFile.php%3FF%3DF_3yjWbA5H9yDVMpg)

Narrative: Naomi Seibt, a German climate change denier, reverses her opinion against climate change and calls for public awareness of the environment crisis on an interview.

Real: [https://cmu.ca1.qualtrics.com/CP/File.php?F=F_7V4dE3qHpQPARwy](https://protect2.fireeye.com/v1/url?k=e4cd9699-bb56ae65-e4cdb8ce-ac1f6b733ff0-bca2a3eeda28762e&q=1&e=1d7ba9fb-5776-4722-a283-a5c6ced696a8&u=https%3A%2F%2Fcmu.ca1.qualtrics.com%2FCP%2FFile.php%3FF%3DF_7V4dE3qHpQPARwy)

Narrative: Naomi Seibt, a German climate change denier, speaks on her journey to climate realism and the state of science.

**References**

1. J T. Dobber, N. Metoui, D. Trilling, N. Helberger, and C. de Vreese, Do (microtargeted) deepfakes have real effects on political attitudes? *The International Journal of Press/Politics* **26**, 1 (2020).
2. S. Fan, R. Wang, T.-T. Ng, C. Y.-C. Tan, J. S. Herberg, and B. L. Koenig, Human perception of visual realism for photo and computer-generated face images. *ACM Transactions on Applied Perception (TAP)* **11**, 2 (2014).
3. A. Leiserowitz, N. Smith, and J. R. Marlon, American teens’ knowledge of climate change. *Yale University. New Haven, CT: Yale Project on Climate Change Communication* **5** (2011).
4. S. K. Yeo, M. A. Xenos, D. Brossard, and D. A. Scheufele, Selecting our own science: How communication contexts and individual traits shape information seeking. *The ANNALS of the American Academy of Political and Social Science* **658**, 1 (2015).
5. H. Cheng and J. Gonzalez-Ramirez, Trust and the media: Perceptions of climate change news sources among US college students. *Postdigital Science and Education* **3**, 1 (2021).
6. K. Gay, J. Torous, A. Joseph, A. Pandya, and K. Duckworth, Digital technology use among individuals with schizophrenia: Results of an online survey. *JMIR Mental Health* **3**, 2 (2016).
7. K.-S. Kim, S.-C. J. Sin, and T.-I. Tsai, Individual differences in social media use for information seeking. *The Journal of Academic Librarianship* **40,** 2 (2014).

**Appendix C: Creation of Factors from Survey Items**

When analyzing questions 5a (frequency of using various information sources to learn about climate change), 5b (trust in those information sources), and 6b (frequency of use of various social media platforms) in the survey detailed in Appendix B, we sought to investigate the potential number of underlying constructs within each question. For example, in question 5a we sought to investigate whether the different news sources were truly distinct or clustered into a smaller number of underlying types of news sources. To do so, we performed exploratory factor analysis on the response options of each question. Specifically, we employ principal components analysis and analyze the eigenvalues of potential factors. As is common practice, we retain factors whose eigenvalue is greater than 1^1^. We choose to employ exploratory factor analysis, and not confirmatory factor analysis, because we did not have a firm hypothesis for the number of constructs that should underlie each question. Rather, we sought to explore the possible number of constructs. In each case, only one underlying construct was found. We then rotated the matrix and predicted the factor score that was used in the analysis.

**Reference**

1. R.K. Henderson and J.K. Roberts, Use of exploratory factor analysis in published research: Common errors and some comment on improved practice. *Educational and Psychological Measurement* **66**, 3 (2006).
